# LncRNA *Airn* alleviates diabetic cardiac fibrosis by inhibiting activation of cardiac fibroblasts via a m6A-IMP2-p53 axis

**DOI:** 10.1186/s13062-022-00346-6

**Published:** 2022-11-16

**Authors:** Tingwei Peng, Mingchuan Liu, Lang Hu, Dong Guo, Di Wang, Bingchao Qi, Gaotong Ren, Chenchen Hu, Feng Zhang, Hyung J. Chun, Liqiang Song, Jianqiang Hu, Yan Li

**Affiliations:** 1 Department of Cardiology, Tangdu Hospital, The Fourth Military Medical University, Xi’an, 710038 People’s Republic of China; 2 Department of Immunology, The Fourth Military Medical University, Xi’an, 710032 Shaanxi People’s Republic of China; 3 State Key Laboratory of Ophthalmology, Zhongshan Ophthalmic Center, Sun Yat-Sen University, Guangdong Provincial Key Laboratory of Ophthalmology and Visual Science, Guangzhou, 510060 People’s Republic of China; 4 Yale Cardiovascular Research Center, Section of Cardiovascular Medicine, Department of Internal Medicine, Yale University School of Medicine, New Haven, CT 06511 USA; 5 Department of Pulmonary and Critical Care Medicine, Xijing Hospital, The Fourth Military Medical University, Xi’an, 710038 People’s Republic of China

**Keywords:** Diabetic cardiomyopathy, Fibrosis, LncRNA-*Airn*, m6A modification, P53, Fibroblast proliferation

## Abstract

**Background:**

Cardiac fibrosis is a leading cause of cardiac dysfunction in patients with diabetes. However, the underlying mechanisms of cardiac fibrosis remain unclear. This study aimed to investigate the role of the long non-coding RNA (LncRNA) *Airn* in the pathogenesis of cardiac fibrosis in diabetic cardiomyopathy (DCM) and its underlying mechanism.

**Methods:**

Diabetes mellitus (DM) was induced in mice by streptozotocin injection. An intramyocardial adeno-associated virus (AAV) was used to manipulate *Airn* expression. The functional significance and underlying mechanisms in DCM fibrosis were investigated both in vitro and in vivo.

**Results:**

Diabetic hearts showed a significant impairment in cardiac function, accompanied by obviously increased cardiac fibrosis. Interestingly, lncRNA *Airn* expression was significantly decreased in both diabetic hearts and high glucose (HG)-treated cardiac fibroblasts (CFs). AAV-mediated *Airn* reconstitution prevented cardiac fibrosis and the development of DCM, while *Airn* knockdown induced cardiac fibrosis phenotyping DCM. As in vitro, *Airn* reversed HG-induced fibroblast-myofibroblast transition, aberrant CFs proliferation and section of collagen I. In contrast, *Airn* knockdown mimicked a HG-induced CFs phenotype. Mechanistically, we identified that Airn exerts anti-fibrotic effects by directly binding to insulin-like growth factor 2 mRNA-binding protein 2 (IMP2) and further prevents its ubiquitination-dependent degradation. Moreover, we revealed that *Airn*/IMP2 protected p53 mRNA from degradation in m6A manner, leading to CF cell cycle arrest and reduced cardiac fibrosis. As a result, ablation of p53 blunted the inhibitory effects of *Airn* on fibroblast activation and cardiac fibrosis.

**Conclusions:**

Our study demonstrated for the first time that *Airn* prevented the development of cardiac fibrosis in diabetic heart via IMP2-p53 axis in an m6A dependent manner. LncRNA *Airn* could be a promising therapeutic target for cardiac fibrosis in DCM.

**Graphical abstract:**

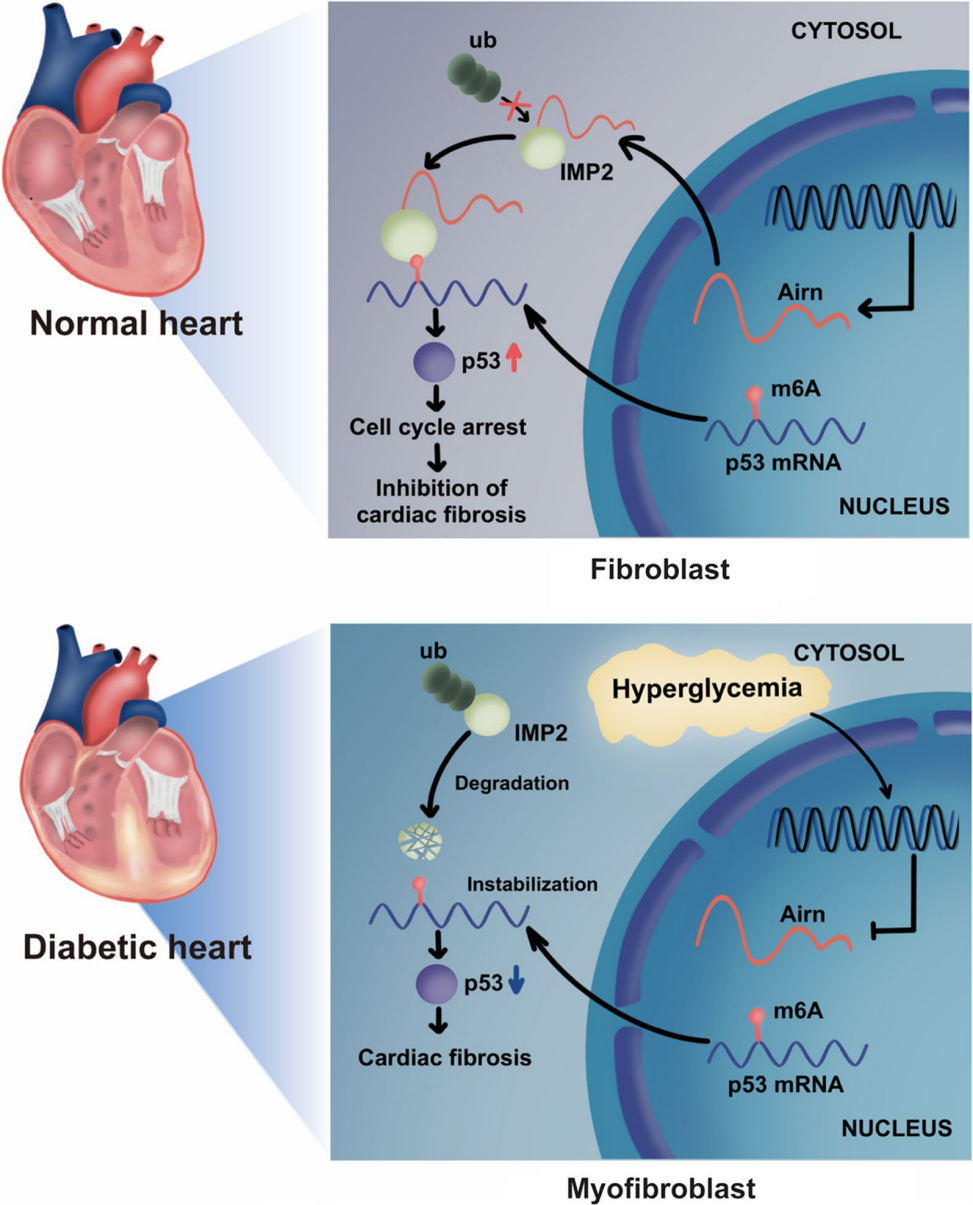

**Supplementary Information:**

The online version contains supplementary material available at 10.1186/s13062-022-00346-6.

## Background

According to the International Diabetes Federation (IDF), worldwide nearly 463 million suffer from diabetes and this number is projected to reach 548 million by 2045 [1]. Diabetic cardiomyopathy (DCM) is a vital complication of long-term diabetes mellitus, characterized in the early stage by cardiac diastolic dysfunction and later by heart failure and cardiac death, in the absence of hypertension, valvular heart disease and coronary artery disease [2]. Extensive studies suggest that myocardial fibrosis, characterized by excessive deposition of extracellular matrix, is responsible for impaired left ventricular compliance and cardiac diastolic function; it is the major pathological feature of DCM [3–7]. However, the mechanism underlying cardiac fibrosis in DCM remains unclear. Previous studies suggest that cardiac fibroblast (CFs) initiate cardiac fibrosis which is activated by multiple pathological stimuli, thus resulting in dysregulated proliferation, fibroblast-to-myofibroblast transition (FMT), and excess production of extracellular matrix [8]. Since activation of CFs is irreversible, targeting cardiac fibrosis currently poses a challenge.

N6-methyladenosine (m6A) is a reversible and dynamic biological process mediated by methyltransferases (writers), demethylases (erasers), and m6A-binding proteins (readers). These serve crucial functions in the m6A modification of mRNA in the progression of cardiovascular diseases (CVDs), including heart failure, myocardial infarction, hypertrophy, and diabetic cardiomyopathy [9–15]. Previous findings demonstrate the m6A-methylation profile of the heart tissues of diabetic mice and suggest that differential m6A-modified mRNAs play an important role in cardiac fibrosis [16]. In general, methyltransferases or ‘writers’ add N6-methyladenosine to specific mRNAs which can be reversed by demethylases or ‘erasers’. METTL3 and FTO reportedly regulate cardiac fibrosis by mediating the activation of fibroblasts and cardiomyocyte contractile by m6A modifications after myocardial infarction [10, 17]. Insulin-like growth factor 2 mRNA-binding protein2 (IMP2), IGF2BP2, a novel m6A reader, can promote translation and stability of mRNA and protein expression. Previous investigations show that IMP2 can promote tumorigenesis by increasing the expression of oncogenes, including MYC, SOX2, and HMGA1 in an m6A-dependent manner [18–20]. Additionally, IMP2 directly contributes to the progression of diabetes [21–23], whereas the role of IMP2 in DCM fibrogenesis remains unknown.

lncRNAs are transcripts with a sequence length greater than 200 nucleotides; these lack protein-coding potential and are closely related to CVDs. This may be partly attributed to their interaction with m6A modification enzymes [24–26]. The lncRNA-*Airn* is imprinted paternally and is located in the antisense orientation to the imprinted insulin growth factor 2 receptor (Igf2r) gene on chromosome 17; thus it regulates the expression of the protein-coding genes in its vicinity, including Igf2r, Slc22a2, and Slc22a3 [27–30]. *Airn* is reportedly associated with the development of several diseases, such as hepatocellular carcinoma, alcoholic fatty liver disease, breast cancer, and diabetic nephropathy as it can regulate cellular apoptosis, migration, and mitophagy [31–34]. Additionally, *Airn* binds to IMP2, thus controlling the translation of several genes involved in myocardial infarction. *Airn* silencing results in enhanced cardiomyocyte death and cellular vulnerability to stress [35]. Considering the high expression of *Airn* in the normal myocardial tissue and its function in cell viability, we wondered the role of *Airn* in DCM fibrosis and its mechanism of m6A modification.

In this study, we confirmed that myocardial fibrosis was significantly higher in heart tissues of diabetic mice, while the expression of *Airn* and IMP2 decreased markedly in the cardiac fibrotic tissues. Reconstitution of *Airn* in mice using AAV9-Lnc-*Airn* could alleviate myocardial fibrosis and cardiac dysfunction after three months of STZ injection. Mechanistically, in-vivo and in-vitro evidence indicated that *Airn* could protect IMP2 from degradation and restore its recognition of m6A-modified p53 mRNA, along with promoting its stabilization, thus ultimately preventing high-glucose-induced CF activation. Taken together, our results suggested that *Airn* was an essential co-factor for epigenetic modulation and that it may serve as a new therapeutic target for cardiac fibrosis in DCM.

## Materials and methods

### Animals

All animal experiments were performed following the Guidelines for the Care and Use of Laboratory Animals, Eighth Edition (2011). C57BL/6J wild-type (WT) mice were provided by Air Force Medical University Animal Center, Xi’an, China. The mice were maintained in a specific pathogen-free (SPF) environment.

### Isolation and culture of primary neonatal and adult mice CFs

CFs were extracted from the neonatal mice (within three days of their birth), as described previously [36]. Briefly, the neonatal mice heart tissues were cut into small sections and washed twice with PBS to remove blood. Subsequently, the sections were digested with collagenase type I (1 mg/mL, Thermo Fisher Scientific, Waltham, MA, USA). The digestion process was terminated by the addition of the complete medium supplement. After adhesion for 1.5 h, cardiomyocytes were removed from cell plates. The purified and isolated CFs were cultured in a normal-glucose medium for 24 h. After 24 h incubation, the dose of 33 mmol/l glucose were selected for high glucose treatment. For NG group, to control the osmotic pressure, an equal amount of mannitol (27.5 mmol/l) was added to DMEM containing 5.5 mmol/l glucose. CFs were collected after 24 h incubation.

CFs were separated from adult mice as described previously [37]. Briefly, the hearts were minced, pooled, and digested in Collagenase digestion cocktail according to previous protocol. After CFs purification via the differential adhesion, CFs were expanded in DMEM with 10% fetal bovine serum and 1% penicillin streptomycin solution.

### Model of diabetic mouse

Eight-week-old male mice received consecutive intraperitoneal injections of streptozotocin for five days (Sigma-Aldrich, St. Louis, MO, USA) (50 mg·kg-1 STZ in 0.1 M citrate buffer solution, pH 4.5) to induce diabetes. After seven days, the blood was drawn from the tail vein to measure the random blood glucose levels using a glucometer (Yuyue, China). Qualified diabetic mice were confirmed and enrolled if they exhibited hyperglycemia (> 16.7 mmol/l). The same batch of mice was treated with citrate buffer solution only in the control group.

### Echocardiography

Echocardiographic images were obtained in the M-mode on the Vevo 2100 echocardiography system (VisualSonics, Toronto, ON, Canada). Thus, the left ventricular systolic and diastolic motion profiles were obtained as described previously [38]. All the digital images were analyzed using the Vevo 2100 software.

### Western blotting (WB)

Total proteins were extracted from heart tissues and CFs using RIPA buffer (Beyotime, China). Subsequently, the samples were incubated for 30 min at 4 °C and centrifuged at 12,000*g* for 10 min at 4 °C. Protein samples were separated on SDS-PAGE and separated proteins were transferred onto polyvinylidene difluoride (PVDF) membranes, following which they were blocked with a 5% non-fat milk solution. After incubation with primary antibodies overnight at 4 °C, the membranes were incubated with HRP-conjugated secondary antibodies for 1 h at 25 °C. Protein expression was detected using the LabImage software (Bio-Rad, Hercules, CA, USA). The relevant primary and secondary antibodies used against the target proteins are listed in Table 1.

**Table 1:** Primary antibodies

Antibody	Corporation	Catalog number
CDK**2**	Proteintech	60312-1-ig
Cyclin D1	Proteintech	60186-1-ig
CDK4	Proteintech	66950-1-ig
DYKDDDDK-tag	Proteintech	66008-3-ig
p21	Proteintech	10355-1-AP
p53	Proteintech	60283-2-ig
IMP2	Cell signaling technology	#14672
Smooth muscle actin	Proteintech	14395-1-AP
Beta actin	Proteintech	66009-1-ig
Collagen I	Abcam	ab138492
Ubiquitin	Cell signaling technology	#3936

### Quantitative real-time PCR

Total RNA was isolated using the RNAisoPlus (Takara, Japan) kit according to the manufacturer’s protocol. Real time-PCR (RT-PCR) was performed using the PrimeScript™ RT Reagent Kit with gDNA Eraser (Takara, Japan) and SYBR® Premix Ex Taq™ II (Takara, Japan) on the CFX96 real-time PCR detection system (Bio-Rad, Hercules, CA, USA). All primer sequences used in this study are listed in Table 2.

**Table 2:** Sequences of primers used in qPCR analysis

Name of primer	Sequence
Airn-Forward	CTGCTGTTGCTGACCTGTAA
Airn-Reverse	CAGTTACCACGCAGACATCC
Gadd45b-Forward	GGAGACATTGGGCACAACCGAA
Gadd45b-Reverse	CTGCTCTCTTCACAGTAACTGGC
Mapk7-Forward	CAGCCTTCTACATCAGAGTCACC
Mapk7-Reverse	CCTTTGGAGTGCCAGAGAACAC
Smad5-Forward	CAGGAGTTTGCTCAGCTTCTGG
Smad5-Reverse	ACGTCCTGTCGGTGGTACTCTG
Mmp14-Forward	GGATGGACACAGAGAACTTCGTG
Mmp14-Reverse	CGAGAGGTAGTTCTGGGTTGAG
Tcf7l1-Forward	CCTCTCATCACCTACAGCAACG
Tcf7l1-Reverse	CTGGAGACAGTGGGTAATACGG
p53-Forward	AGAGACCGCCGTACAGAAGA
p53-Reverse	CTGTAGCATGGGCATCCTTT
p53-RIP-Forward	TGTCACGCTTCTCCGAAGAC
p53-RIP-Reverse	AGCAACAGATCGTCCATGCA
β-Actin-Forward	CATTGCTGACAGGATGCAGAAGG
β-Actin-Reverse	TGCTGGAAGGTGGACAGTGAGG
IMP2-Forward	GGTGCTATCATCGGGAAGAA
IMP2-Reverse	CTTCAGGAGGACCAGTGATGA

### Assessment after downregulation or upregulation of target genes

For animal experiments, adeno-associated virus 9 carrying Airn (AAV9-Airn), Airn-specific small hairpin RNA (AAV9-shAirn), and the control (AAV9-EV) constructs were obtained from Hanheng Biotechnology (Hanheng Biotechnology Co., Ltd., Shanghai, China). Twelve-week-old mice in each group were intramyocardially injected with the corresponding AAV constructs as reported previously [38].

For cell experiments, downregulation of target genes was performed by transient siRNA transfection, using RNAiMax Transfection Reagent (Invitrogen) according to manufacturer’s protocol. Si-Airn, si-IMP2 and Negative control small interfering RNA (si-NC) were synthesized and designed by GenePharma (Shanghai, China), and the specific siRNA for p53 was purchased from Santa Cruz (Catalog number: sc-29436). For upregulating of target genes, overexpression plasmids from GenePharma Company (Shanghai, China) were used to transfect the cardiac fibroblasts. After 48 h transfection, cell lysates were detected by western blot (WB) or real time-PCR (RT-PCR). The detailed information of siRNA is provided in Table 3.

**Table 3:** The sequences of siRNAs

siRNA	Sequence (5′–3′)
siAirn	Sense: CAAAUGGCGCUCAACUACAdTdT
	Antisense: UGUAGUUGAGCGCCAUUUGdTdT
siIMP2	Sense: GACCAAACGCCAGACGAGAdTdT
	Antisense: UCUCGUCUGGCGUUUGGUCdTdT
sip53	Sense: CCUCCAAGAUGAUGCACAUTT
	Antisense: AUGUGCAUCAUCUUGGAGGTT
Negative control	Sense: UUCUCCGAACGUGUCACGUTT
	Antisense: ACGUGACACGUUCGGAGAATT

### RNA fluorescence in situ hybridization (FISH) and immunofluorescence (IF) staining

RNA-FISH and immunofluorescence co-staining were employed to detect the colocalization of IMP2 protein, Airn, and p53 mRNAs. Airn and p53 mRNA fluorescence was estimated in CFs using the FISH Kit (GenePharma, China). Probes were incubated at 37 °C for 4 h and their sequences designed by GenePharma Company (Shanghai, China). Subsequently, CFs were incubated with anti-IMP2 antibodies overnight at 4 °C. After incubation with secondary antibodies and DAPI, images were obtained with a confocal microscope (Nikon A1R MP + Confocal Microscope; Nikon, Tokyo, Japan). All FISH probes sequences are provided in Table 4.

**Table 4:** FISH probes sequences

Gene	Sequences
Negative control	TGCTTTGCACGGTAACGCCTGTTTT
p53	ACACTCGGAGGGCT + TCACTTGGGC
	ATGG + TAAGGATAGGTCGGCGGT + TC
	CCTCAT + TCAGCTCCCGGAACA + TCTC
Airn	AGGATGTC + TGCGTGGTAAC + TGGCG
	GCTGACC + TGTAAACCAAAC + TGCCGA
	GGACT + TGGGTCAAC + TGGCAGAGTGA
18s	CTGCCTTCCTTGGATGTGGTAGCCGTTTC

### RNA-pulldown assay

Biotin-labeled Airn was synthesized by T7 transcription in vitro with the help of GenePharma Company (Shanghai, China). Total proteins were extracted using the Pierce IP Lysis Buffer (Thermo Fisher Scientific). We used Magnetic RNA–Protein Pull-Down Kit (Thermo Fisher Scientific) for the RNA pull-down assays. First, streptavidin magnetic beads were incubated with labeled RNA overnight at 4 °C. Subsequently, the bead mixture was incubated with cell lysates at 4 °C for 6 h. After washing and elution of the RNA-binding protein complexes, the elutes were detected by WB and silver staining.

### RNA immunoprecipitation (RIP) and m6A-RNA immunoprecipitation assay (Me-RIP)

RIP assay was performed using the Magna RNA-Binding Protein Immunoprecipitation Kit (Millipore). Briefly, cell lysates were prepared in the RIP lysis buffer. After washing the A/G magnetic beads thrice, purified antibodies were incubated with these overnight at 4 °C. Subsequently, targeted RNAs were captured with the A/G magnetic beads-antibodies complexes by incubating on a rotor at room temperature for 4 h. Finally, the RNA mixture was digested with proteinase K and 10% SDS at 55 °C for 30 min. The immunoprecipitated RNA was detected by qRT-PCR analysis. For the Me-RIP, the Magna Me-RIP m6A Kit (Millipore, USA) was used to measure the m6A-methylation rate of p53 following the manufacturer’s protocol. Antibodies and primer sequences used in this experiment are listed in Tables 1 and 2.

### Immunoprecipitation (IP) assay

IP assay was performed using the Pierce Classic Magnetic IP/Co-IP Kit (Thermo Fisher Scientific) according to the manufacturer’s instructions. Anti-IMP2 antibody and anti-FLAG/DYKDDDDK tag were used for the IP assay. Finally, ubiquitination of IMP2 was detected by WB using the anti-ubiquitin antibody.

### Masson, immunofluorescence (IF), and immunohistochemical analyses (IHC)

Mice heart tissues were fixed in 4% paraformaldehyde (pH 7.4) overnight at 4 °C and these paraffin-embedded heart sections (5-mm-thick slices) were used for histological analysis. Interstitial fibrosis was assessed by Masson’s trichrome staining.

Immunohistochemistry was conducted as described previously [38]. Briefly, heart sections were treated with 3% hydrogen peroxide (H_2_O_2_) for 20 min at room temperature to block endogenous peroxidase activity. The sections were incubated with primary antibodies against collagen-1 overnight at 4 °C. After washing with PBS, tissue sections were incubated with horseradish peroxidase (HRP)-conjugated secondary antibody for 1 h. Following immunostaining, the sections were counterstained with hematoxylin and mounted.

To evaluate proliferation and FMT of primary neonatal CFs, immunofluorescence staining for Ki67 and α-SMA was performed for the sections. Briefly, sections were deparaffinized, and rehydrated, following which the endogenous peroxidase activity was blocked. After blocking with normal 5% goat serum (Beyotime, China) for 1 h, sections were incubated overnight with anti-Ki67 and α-SMA antibodies at 4 °C. Subsequently, sections were incubated with a secondary antibody for 1 h at room temperature. Nuclei were stained with DAPI. Images were captured on the EVOS M5000 fluorescence imaging system (Thermo Fisher, USA).

### 5-Ethynyl-2′-deoxyuridine (EdU) assay

The Edu assay was performed using the Edu kit (Beyotime, China). After incubation with Edu working solution (1:1000) for 2 h at 37 °C, the cells were fixed with 4% paraformaldehyde for 15 min and treated with 0.3% Triton X-100 for 30 min. Subsequently, the click reaction solution was added to cells for 30 min, following which the nuclei were stained with Hoechst 33342 for 10 min. EVOS M5000 fluorescence imaging system (Thermo Fisher, USA) was used to examine the positively labeled proliferative cells.

### CCK8 assay

The CCK8 Kit (Dojindo, Shanghai, China) was used to detect cellular proliferation following the manufacturer’s protocol. Briefly, cells were seeded in 96-well plates at a density of 1 × 10^4^ cells per well. After 24 h of transfection, 100 μL of the CCK8 solution (10 μL CCK8:100 μL medium) was added per well and cells were incubated for 2 h at 37 °C. Finally, absorbance was measured at 490 nm using an Epoch microplate reader (BioTek, USA).

### Cell cycle analysis

The cell cycle was analyzed by flow cytometry. After collecting cells post trypsinization, the cells were centrifuged and fixed overnight at 4 °C in 75% ice-cold ethanol. Cells were stained with 1 mL propidium iodide (PI) solution (20 μg/mL PI in the presence of RNase-A) for 30 min. Samples were analyzed on the BD FACSCanto™ II flow cytometer (BD Biosciences) and processed using the Cell Fit Cell Analysis program (Becton Dickinson Immunocytometry Systems, NJ, USA).

### Evaluating mRNA and protein stability

To assess mRNA and protein stability, cells were treated with 5 μg/mL actinomycin D (Act-D, MCE, China) and 20 μg/mL cycloheximide (CHX, Merck, Germany). Cells were harvested at specific time points. RNA and proteins were isolated for RT-PCR and WB, respectively, to analyze their half-lives. Detailed calculations are described previously [39].

### TGF-β1 or disitertide treatment

100 ng/mL TGF-β1 (MedChemExpress, USA) was dissolved in DMEM to a final concentration of 0.2 ng/mL. 1 mM Disitertide (MedChemExpress, USA) was diluted in DMEM at a final concentration of 10 μmol/L. After incubation with TGF-β1 or disitertide for 12 h, the CFs were harvested for investigation.

### Statistical analyses

All data in this study were analyzed using the GraphPad Prism v 8.0 Software (San Diego, CA, USA). Between-group comparisons were performed using Student's t-test. One-way ANOVA was performed for comparisons among multiple groups. Data were expressed as mean ± SEM. *p* value < 0.05 was considered statistically significant.

## Results

### *Airn* expression were downregulated in diabetic hearts, while overexpression of *Airn* ameliorates diabetes-induced cardiac dysfunction

STZ were used to induce diabetes in mice. As shown in Additional file 2: Figure S1A&B, intraperitoneal injections of STZ successfully induced diabetic mellitus (DM) in the mice, which was confirmed by significantly elevated blood glucose and decreased body weight. Cardiac function were determined by echocardiographic analysis. As shown in Additional file 2: Figure S1C-G, mice with DM exhibited a significant impairment in cardiac function, as evidenced by decreased LVEF and LVFS. To explore whether *Airn* was involved in diabetic cardiac fibrosis, cardiac fibrosis indicators and *Airn* expression were determined in control and diabetic hearts. Compared with the control heart, diabetic hearts exhibited significant cardiac fibrosis, as evidenced by elevated collagen I content and increased Masson staining positive area percentage (Additional file 2: Fig. S1H-J). In contrast, *Airn* expression in diabetic hearts decreased gradually as DM progressed (Additional file 2: Fig. S1K). Moreover, RNA decay assays and qRT-PCR showed decreased expression and stability of Airn in neonatal mice cardiac fibroblasts treated with high-glucose (HG)-treatment (Additional file 2: Fig. S1L-N). The down-regulated expression of Airn was also found in cardiomyocytes (Additional file 2: Fig. S1O). These data suggested a strong linkage between *Airn* inhibition and cardiac fibrosis in diabetic mice.

**Fig. 1:**
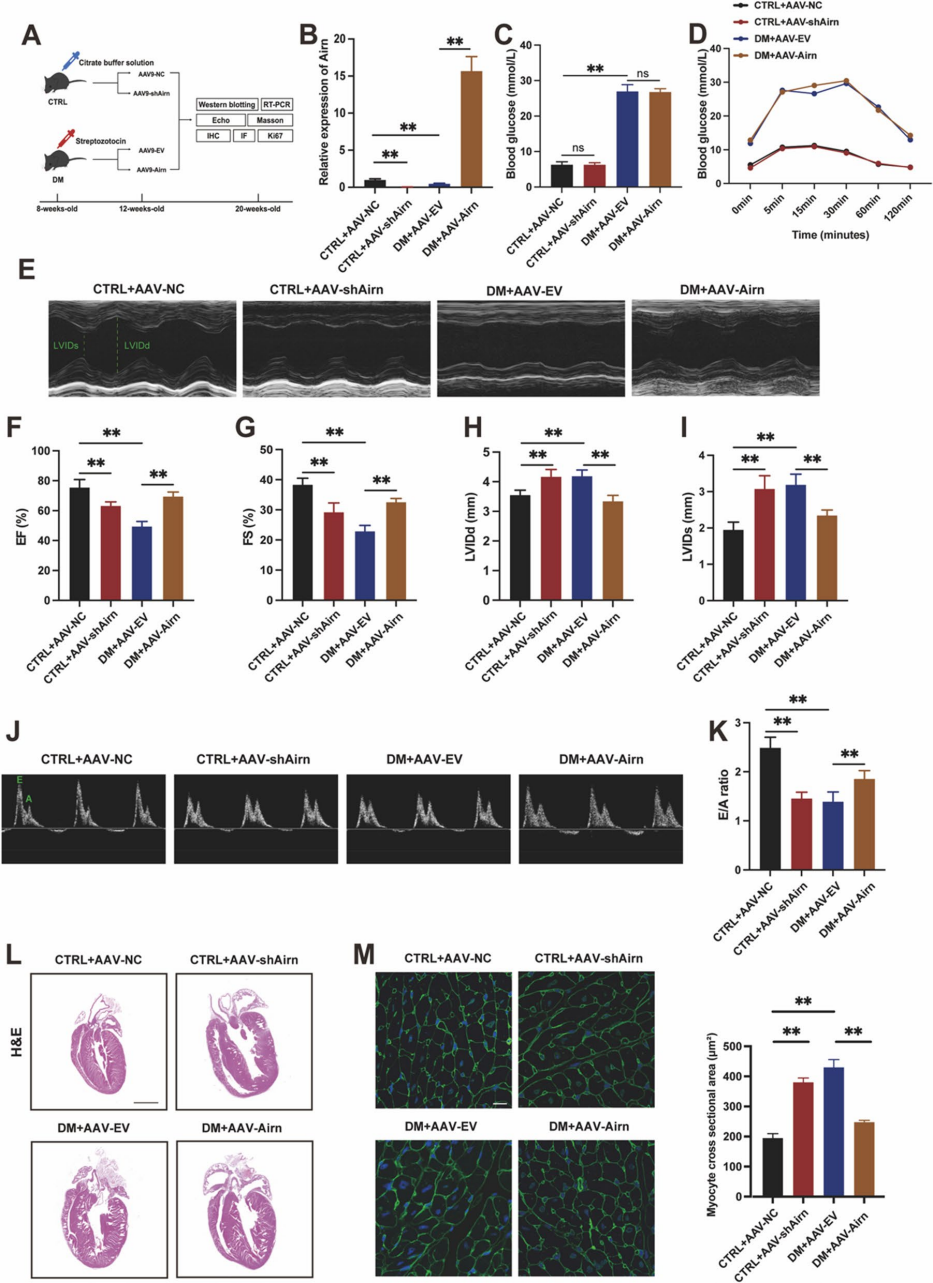
Airn expression were downregulated in diabetic hearts, while overexpression of Airn ameliorates diabetes-induced cardiac dysfunction. **A** Schematic diagram of the experimental protocol. **B** qRT-PCR analysis of Airn expression in CTRL and DM hearts by intramyocardially AAV-9 virus injection. **C** Random blood glucose levels. **D** IPGTT glucose levels. **E** Representative M-mode echocardiography images in CTRL and DM hearts by intramyocardially AAV-9 virus injection. **F**–**I** Echocardiography data analysis. **J** Representative Doppler echocardiography images. **K** E/A ratio. **L** Representative images of the morphologies of the hearts stained by hematoxylin and eosin staining; Scale bar = 200 mm. **M** Representative images of wheat germ agglutinin staining and quantitative analysis of the cross-sectional area of cardiomyocytes. Scale bar = 20 μm. Data are presented as means ± SEM. *p < 0.05; **p < 0.01. n = 6 mice

Next, we aimed to determine whether *Airn* could protect against cardiac fibrosis in the diabetic cardiomyopathy (DCM) model. Gain-of-function studies were performed by employing recombinant adeno-associated virus (AAV)9-*Airn* to up regulate *Airn* expression in diabetic heart through intramyocardial injection (Fig. 1A). After eight weeks of AAV injection, *Airn* expression was detected in heart tissues by polymerase chain reaction (PCR), and results showed successful virus injection with high efficiency (Fig. 1B). Random blood glucose tests and intraperitoneal glucose tolerance test (IPGTT) glucose indicated that *Airn* overexpression or silencing had no effect on blood glucose (Fig. 1C and D). Overexpression of *Airn* alleviated cardiac dysfunction in diabetic mice, as evidenced by significant increases in left ventricular ejection fraction (LVEF) and left ventricular fractional shorting (LVFS), as well as decreases in left ventricular systolic internal dimension and left ventricular diastolic internal dimension (LVIDd) (Fig. 1E–I). Furthermore, Doppler echocardiography showed that *Airn* reconstitution also alleviated diastolic dysfunction in DM mice with an enhanced E/A ratio (Fig. 1J and K). Cardiac hypertrophy is an important structural feature of pathology in DCM [40]. Compared with the control group, DM mice showed significant cardiac hypertrophy as evidenced by hematoxylin, eosin, and wheat germ agglutinin staining (Fig. 1L and M). DM-induced cardiac hypertrophy was efficiently ameliorated in the hearts of DM mice receiving AAV-*Airn*. Taken together, these data indicate that overexpression of *Airn* could ameliorate diabetes-induced cardiac dysfunction. Additionally, we investigated whether cardiac function was perturbed by the loss of *Airn* function in wild-type (WT) mice receiving AAV-sh*Airn*. Notably, WT mice with *Airn* knockdown displayed impaired systolic and diastolic function (Fig. 1E–K) and cardiac hypertrophy (Fig. 1L and M) as compared with control mice. Collectively, we observed the downregulation of *Airn* both in diabetic mice heart tissues and HG-treated CFs and found that *Airn* is an essential LncRNA for DM-induced cardiac dysfunction.

### Overexpression of *Airn* protects against diabetes-induced cardiac fibrosis, while *Airn* knockdown leads to cardiac fibrosis in the normal heart

**Fig. 2:**
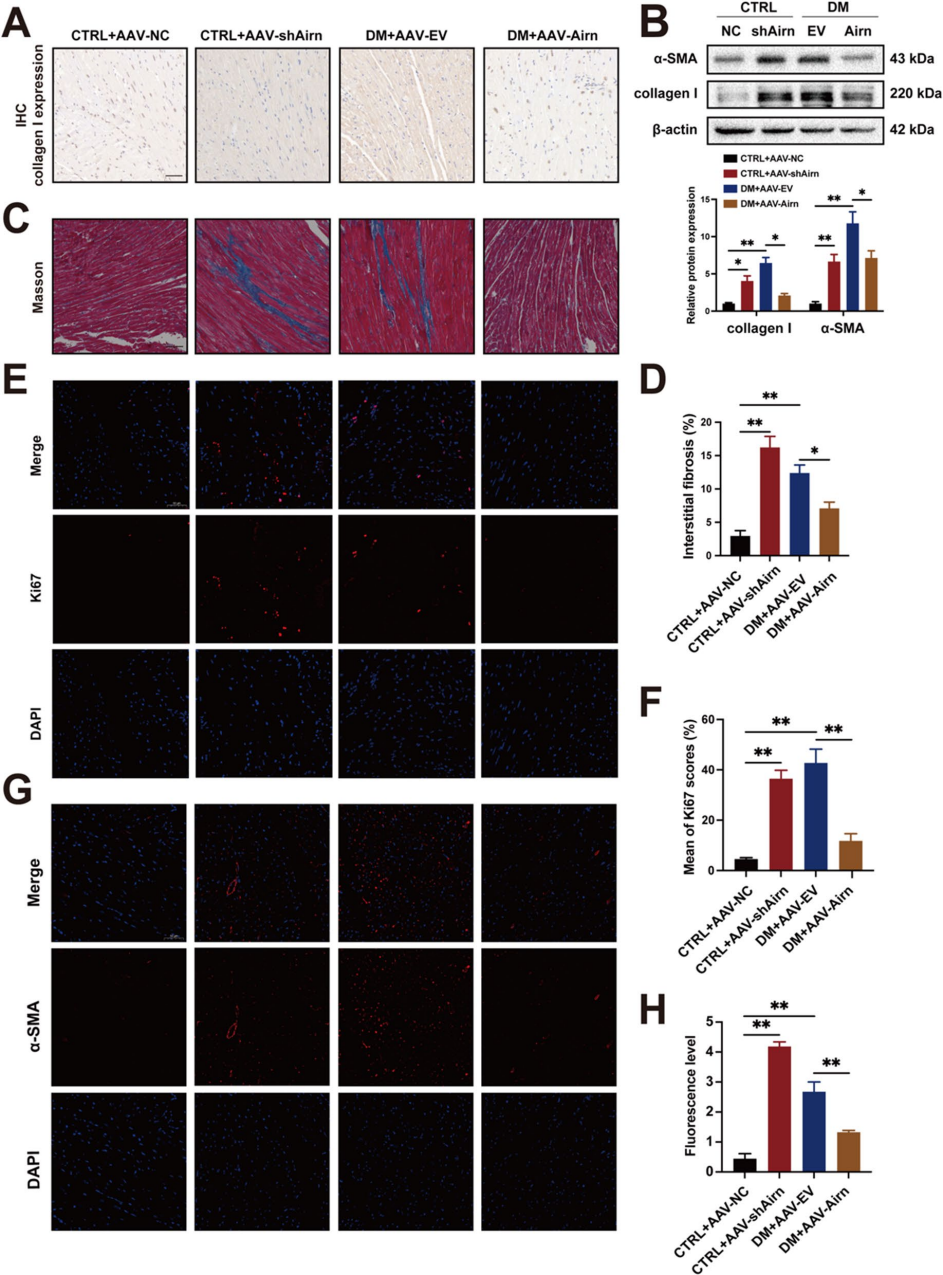
Overexpression of Airn protects against diabetes-induced cardiac fibrosis, while Airn knockdown leads to cardiac fibrosis in the normal heart. **A** Representative images of immunohistochemical staining of collagen I in CTRL and DM hearts by intramyocardially AAV-9 virus injection; Scale bar = 50 μm. **B** Representative blot images and quantitative analysis of collagen I and α-SMA expression in CTRL and DM hearts by intramyocardially AAV-9 virus injection. **C** Representative images of Masson’s trichrome staining in CTRL and DM hearts by intramyocardially AAV-9 virus injection; Scale bar = 50 μm. **D** The quantitative analysis of Masson’s trichrome staining. **E**, **F** Ki67 assay by double staining with DAPI (blue) and Ki67 (red) detected proliferative cells in CTRL and DM hearts by intramyocardially AAV-9 virus injection; The quantification of Ki67-positive cells is shown; Scale bar = 50 μm. **G**, **H** Representative images of immunofluorescence staining for α-SMA (red) and DAPI (blue) and quantification of the relative cell area of CFs in CTRL and DM hearts by intramyocardially AAV-9 virus injection; Scale bar = 50 μm. Data are presented as means ± SEM. *p < 0.05; **p < 0.01. n = 6 mice

Efficient *Airn* overexpression abrogated DM-induced cardiac fibrosis, as demonstrated by Masson’s staining (Fig. 2C and D). Western blotting and immunohistochemical staining showed that expression of the fibrotic marker, collagen I, was significantly decreased in the heart tissues of diabetic mice after AAV-*Airn* treatment, indicating that *Airn* reduced the secretion of collagen I in diabetic heart tissues (Fig. 2A and B). Because the aberrant proliferation and fibroblast-to-myofibroblast transition (FMT) of CFs are key events during cardiac fibrosis [40], we performed immunofluorescence assays for Ki67 and alpha smooth muscle actin (α-SMA) to detect these two cell phenotypes. Immunofluorescence showed excessive proliferation and FMT of CFs in the hearts of the diabetic mice. Moreover, these phenotypes were significantly attenuated by *Airn* overexpression (Fig. 2E–H). In addition, downregulation of *Airn* augmented the production of collagen I, proliferation, and FMT, and further exacerbated myocardial fibrosis in non-diabetic mice compared to that in CTRL mice. Taken together, these results indicate that *Airn* is an important negative regulator of fibrogenesis induced by HG and is a potential therapeutic target for the treatment of DCM fibrosis.

### *Airn* attenuates HG-induced CF proliferation and FMT.

**Fig. 3:**
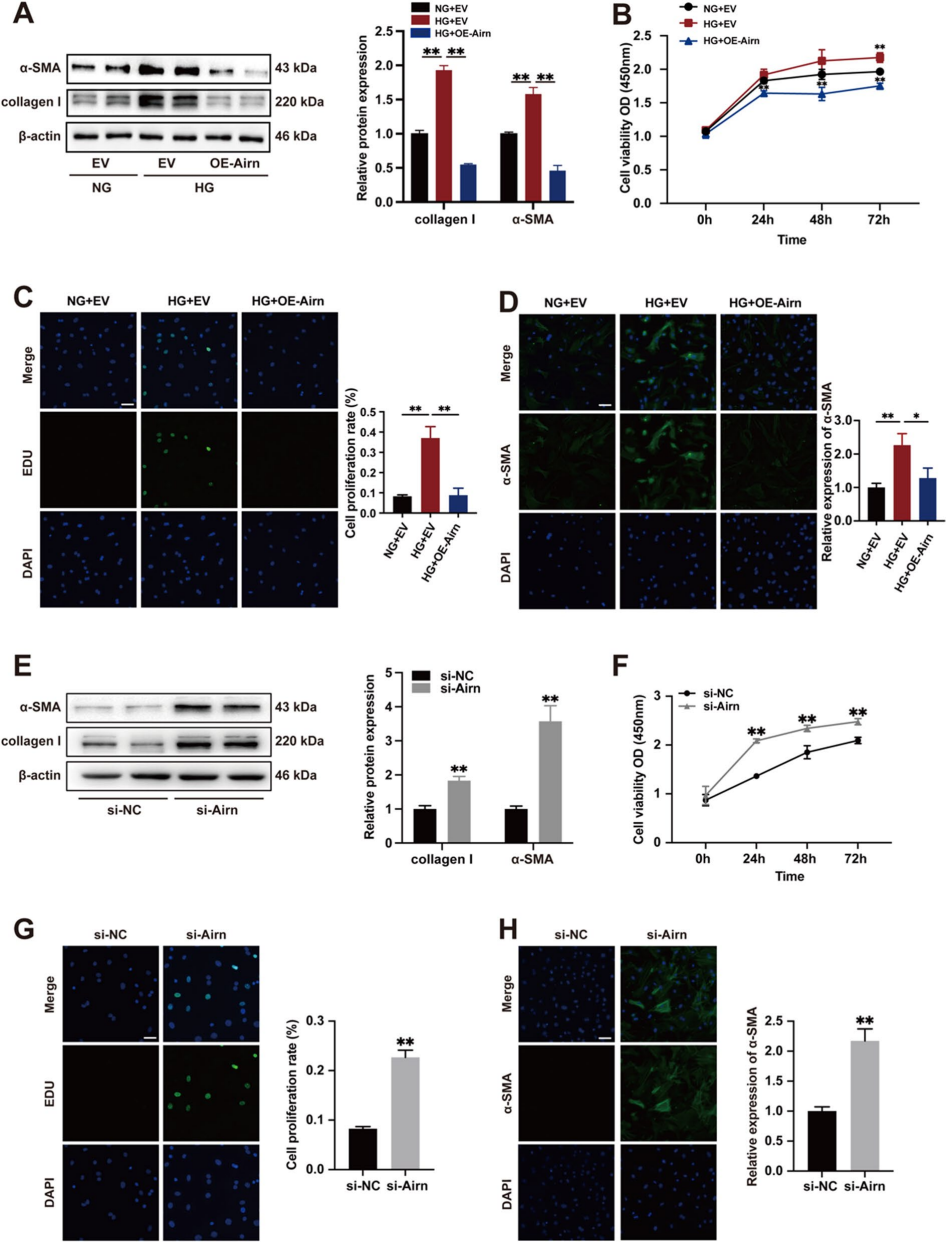
Airn attenuates HG-induced CFs proliferation and FMT. **A**, **E** Representative blot images and quantitative analysis of collagen I and α-SMA expression in CFs. **B**, **F** CCK-8 assay showed the proliferation of CFs. **C**, **G** Representative images of immunofluorescence staining for EdU (green) and DAPI (bule) and quantification of the proliferative cells; Scale bar = 50 μm. **D**, **H** Representative images of immunofluorescence staining for α-SMA (green) and DAPI (blue) and quantification of the relative cell area of CFs; Scale bar = 50 μm. Data are presented as means ± SEM. **p < 0.01. n = 3 wells

We evaluated the function of *Airn* in cardiac fibrosis in vitro. CFs were treated with HG medium to mimic diabetic conditions. As expected, HG treatment alone upregulated α-SMA and collagen I expression (Fig. 3A). However, *Airn* overexpression blocked the HG-induced expression of α-SMA and collagen I (Fig. 3A). Using the CCK8 assay, we found that elevated *Airn* significantly reduced the absorbance of HG-treated CFs compared to CFs treated with HG only, which was consistent with the in vivo results (Fig. 3B). EDU immunofluorescence staining also confirmed that the percentage of EDU-positive cells was significantly lower in *Airn*-overexpressing CFs (Fig. 3C). Meanwhile, immunostaining of α-SMA indicated that upregulated *Airn* levels blunted HG-induced morphological changes in FMT (Fig. 3D).

To investigate whether the loss of *Airn* is essential for CF activation independent of glucose level changes, we used siRNA-*Airn* to suppress the expression of *Airn* in CFs treated with normal glucose (NG) medium. Western blotting revealed that *Airn* knockdown enhanced α-SMA and collagen I expression (Fig. 3E). CCK8 and EDU assays indicated that si-*Airn* treatment accelerated the proliferation of CFs (Fig. 3F and G). As shown by α-SMA immunofluorescence, downregulation of *Airn* activated the FMT of CFs under NG conditions (Fig. 3H). We also found that Airn downregulation also played the fibrotic role *in* cardiac fibroblasts isolated from hearts of CTRL + AAV9-shAirn mice (Additional file 6: Fig. S5B, D, E, H and I). Thus, we concluded that *Airn* plays an essential role in maintaining the normal form and function of CFs in vivo.

### *Airn* exerts its anti-fibrotic effects by directly binding to insulin-like growth factor 2 mRNA-binding protein 2 (IMP2)

**Fig. 4:**
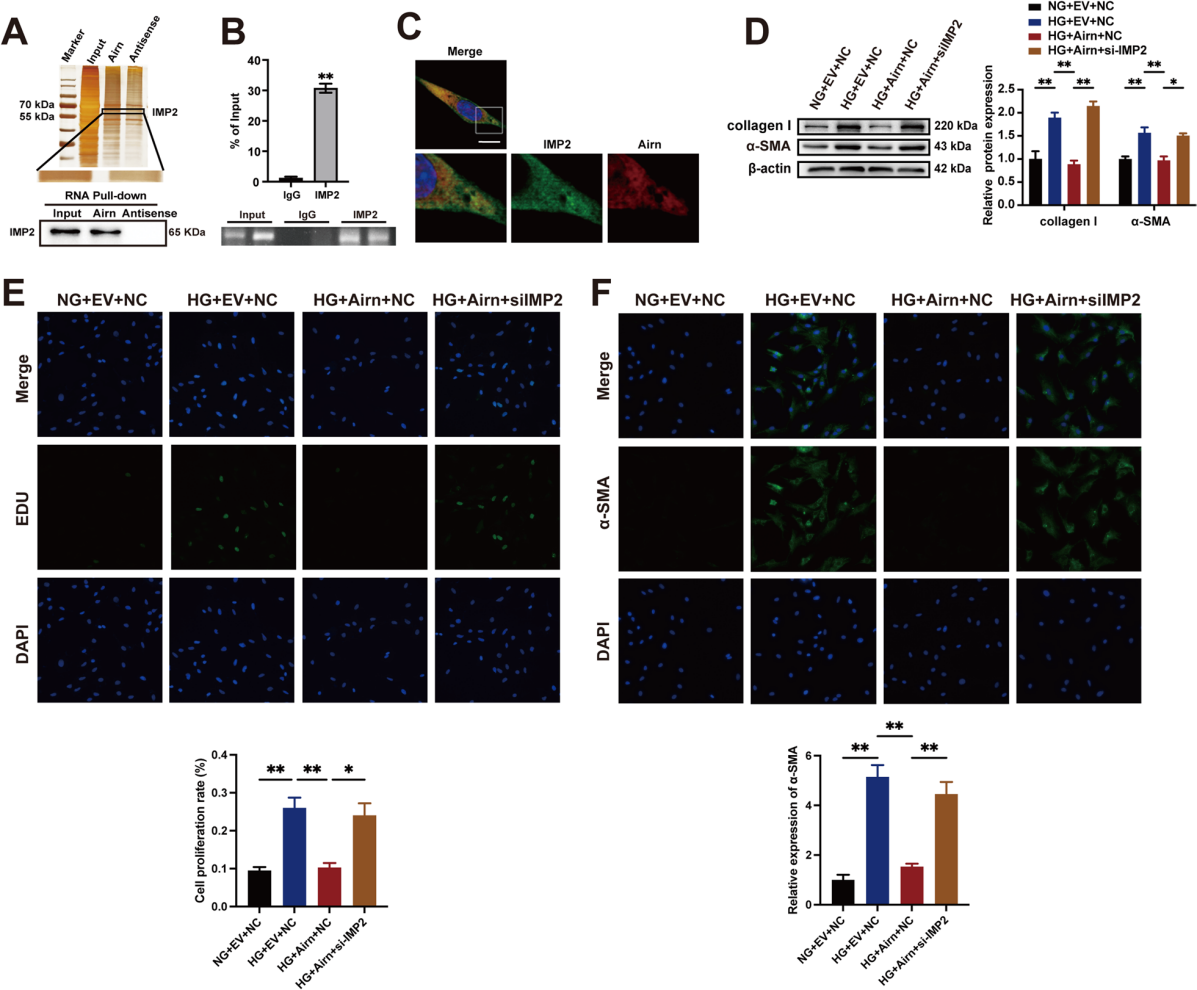
Airn exerts its anti-fibrotic effects by directly binding to insulin-like growth factor 2 mRNA-binding protein 2 (IMP2). **A** IMP2 was pulled down by biotin-labeled sense Airn but not Airn anti-sense RNA in CFs. **B** RIP-qPCR showed the binding between Airn and IMP2 using anti-IMP2 antibody compared with the IgG antibody. **C** RNA FISH and immunofluorescence co-staining was employed to detect the colocalization of IMP2 protein (green), Airn (red) and DAPI (blue); upper scale bar = 20 μm, lower scale bar = 5 μm. **D** Representative blot images and quantitative analysis of collagen I and α-SMA expression in CFs. **E** Representative images of immunofluorescence staining for EdU (green) and DAPI (bule) and quantification of the proliferative cells; Scale bar = 50 μm. **F** Representative images of immunofluorescence staining for α-SMA (green) and DAPI (blue) and quantification of the relative cell area of CFs; Scale bar = 50 μm. Data are presented as means ± SEM. *p < 0.05; **p < 0.01. n = 3 wells

To identify the molecular mechanisms underlying the regulation of DCM fibrosis by *Airn*, its downstream molecules were investigated according to previously reported methods. IMP2 is a potential binding partner of *Airn* [35]. RNA pull-down assays showed that IMP2 was directly bound to sense *Airn* and not to the antisense strand in CFs (Fig. 4A). Furthermore, RIP assay confirmed binding between *Airn* and IMP2 (Fig. 4B). Confocal fluorescence imaging analysis revealed colocalization of IMP2 and *Airn* in the cytoplasm, but not in the nucleus (Fig. 4C). IMP2, an RNA-binding protein, binds to a series of mRNAs and regulates several biological processes by influencing their fate [39]. Thus, we speculated that IMP2 may be involved in *Airn*-mediated phenotypic changes in CFs. To test our hypothesis, we transfected CFs with si-IMP2 and *Airn*-overexpressing plasmids. We found that IMP2 knockdown significantly restored the expression of α-SMA and collagen I in CFs with *Airn* overexpression following HG treatment (Fig. 4D). Furthermore, suppression of IMP2 significantly enhanced the proliferative capacity of *Airn*-overexpressing CFs, as indicated by EDU assay (Fig. 4E). α-SMA immunofluorescence also indicated a reversed role of si-IMP2 in the *Airn* overexpression-mediated inhibition of FMT in CFs (Fig. 4F). Taken together, these results show that IMP2 knockdown reversed the inhibitory effect of *Airn* on the proliferation and FMT of CFs.

### *Airn* protects IMP2 from ubiquitin–proteasome-dependent degradation, thus maintaining its stability

**Fig. 5:**
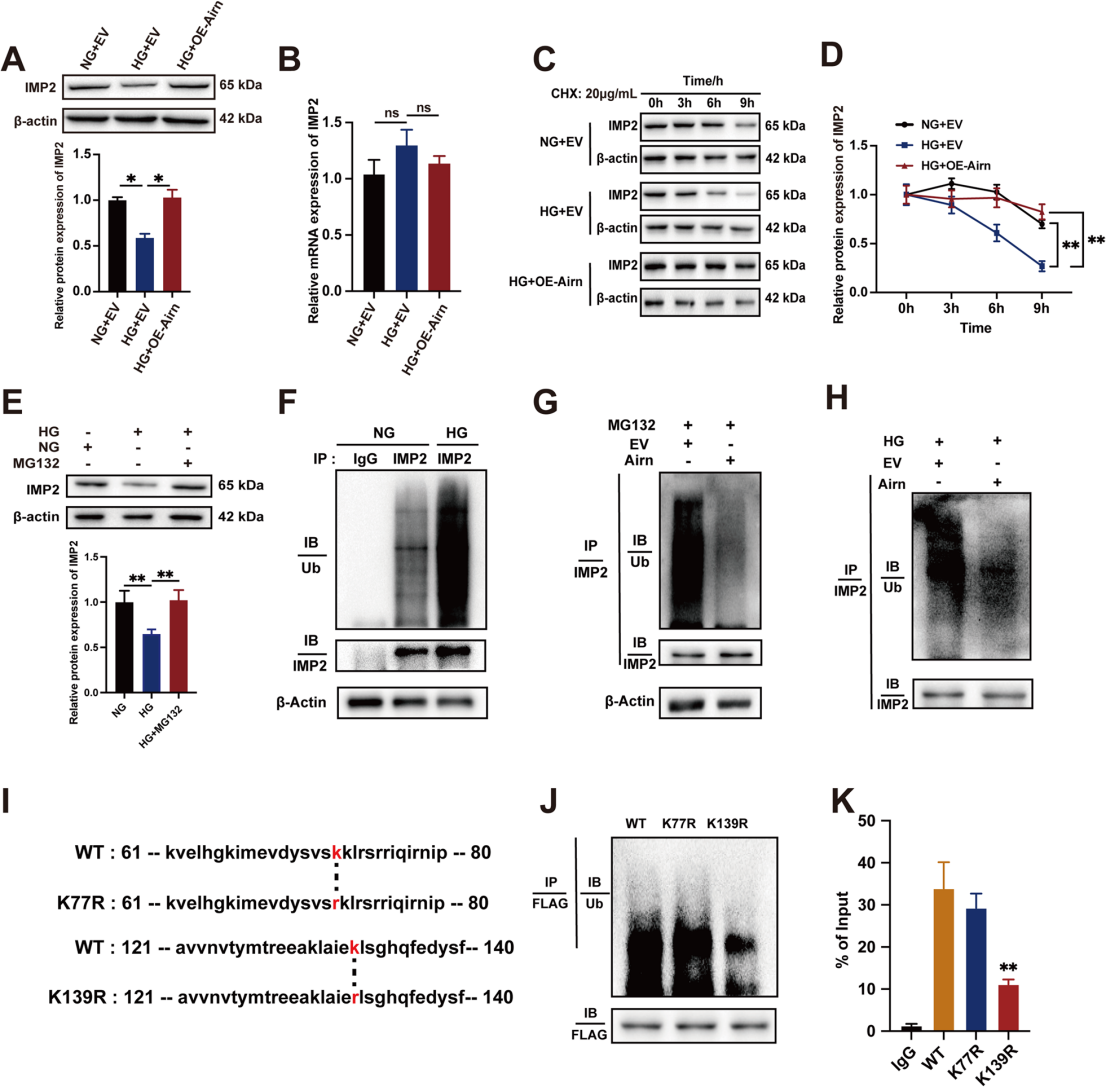
Airn protects IMP2 from ubiquitin–proteasome-dependent degradation, thus maintaining its stability. **A** Representative blot images and quantitative analysis of IMP2 expression in CFs. **B** qRT-PCR analysis of IMP2 in CFs. **C**, **D** CFs were treated with 20 μg/mL cycloheximide (CHX), harvested at indicated points and analyzed by western blotting. **E** After MG132 treatment of CFs cultured in HG-medium, the protein level of IMP2 was detected by western blot. **F**–**H** IP and ubiquitination assays were conducted to investigate the effect of Airn or HG on ubiquitination of IMP2. **I** The sequences of WT, K77R and K139R mutants of IMP2. **J** IP and ubiquitination assays using anti-FLAG antibodies and CFs transfected with plasmids expressing the FLAG-tagged WT, K77R and K139R of IMP2. **K** RIP assays were performed using anti-FLAG antibodies and CFs transfected with plasmids expressing the FLAG-tagged WT, K77R and K139R of IMP2. Data are presented as means ± SEM. *p < 0.05; **p < 0.01. n = 3 wells

Decreased expression of the IMP2 protein was observed in CFs treated with HG, while overexpression of *Airn* significantly increased IMP2 protein levels in HG treatment (Fig. 5A). However, the mRNA levels of IMP2 remained unchanged (Fig. 5B). Next, we assessed the effect of si-*Airn* on IMP2 expression. Depletion of *Airn* resulted in the decline of IMP2 at the protein level, but not at the mRNA level (Additional file 3: Fig. S2A and B). Based on these results, we speculated that IMP2 may be regulated by HG treatment and *Airn* at the post-transcriptional level. To test our hypothesis, we treated CFs with the protein synthesis inhibitor cycloheximide (CHX, 20 μg/mL) and harvested cells at the indicated time points (0, 3, 6, and 9 h). Compared to NG treatment, HG treatment significantly shortened the half-life of the IMP2 protein (Fig. 5C and D). However, *Airn* overexpression extended the protein half-life of IMP2 in CFs cultured in HG, suggesting that *Airn* may be a protector of IMP2 protein stabilization (Fig. 5C and D). To investigate the degradation patterns of IMP2 under HG conditions, CFs were treated with MG132 (0.1 mol/L), a potent proteasome and calpain inhibitor, to inhibit the proteasomal system and thus increase ubiquitin accumulation. MG132 rescued the HG-mediated decrease in IMP2 expression (Fig. 5E), suggesting that degradation of IMP2 may be linked to the accumulation of ubiquitin mediated by HG. Moreover, *Airn* overexpression significantly weakened the enhanced ubiquitination of IMP2 by MG132 (Fig. 5G). Additionally, the ubiquitination of IMP2 was increased significantly in HG-treated CFs as compared to NG-treated CFs, while *Airn* overexpression restrained the effects of HG-induced ubiquitination of IMP2 in CFs (Fig. 5F and H). We also confirmed that the ubiquitination of IMP2 was increased in fibroblasts isolated from the heart receiving AAV9-sh*Airn*-treatment, which led to decreased expression of IMP2 protein subsequently in vivo (Additional file 6: Fig. S5A, B and F).

To locate the binding sites of IMP2 targeted by *Airn*, plasmids expressing FLAG-tagged WT or IMP2 ubiquitin mutants (K77R and K139R) were constructed [18] (Fig. 5I), which were conserved among species (Additional file 1). After transfection with the relevant plasmids, RIP assay showed that the K139 mutation weakened the binding between *Airn* and IMP2 (Fig. 5K). Compared to the WT and K77R, the K139 mutation significantly decreased IMP2 ubiquitination (Fig. 5J). Taken together, the above observations indicate that *Airn* could promote the protein stability of IMP2 by suppressing its ubiquitination through masking of the K139 ubiquitin site and thus slowing IMP2 degradation under HG stimulation.

### *Airn*/IMP2 inhibits HG-induced CF proliferation and activation by maintaining p53-mediated cell cycle arrest.

**Fig. 6:**
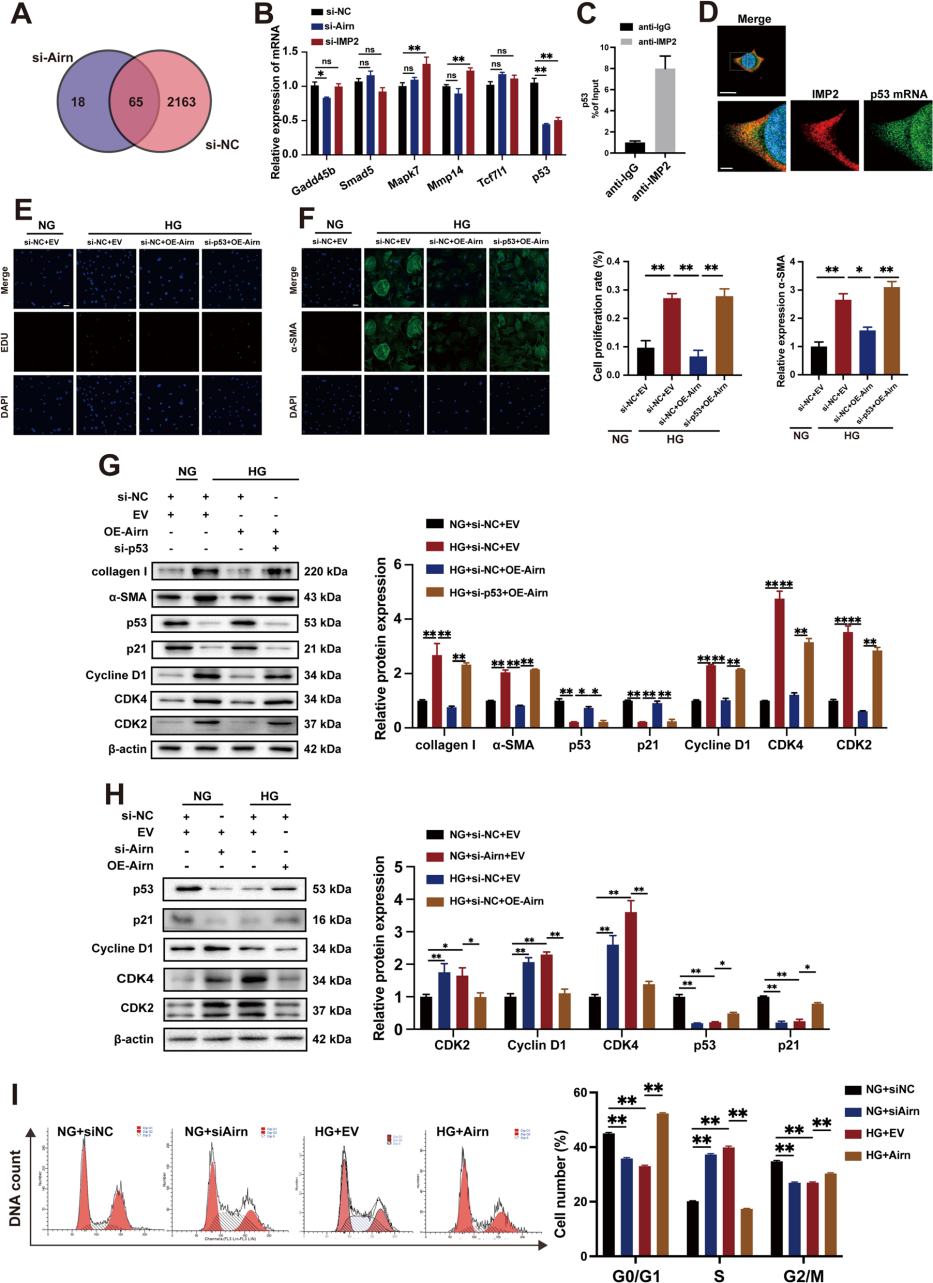
*Airn*/IMP2 inhibits HG-induced CF proliferation and activation by maintaining p53-mediated cell cycle arrest. **A** Venn diagram of IMP2-bound genes following knockdown of Airn obtained from dataset GSE87221 in the GEO database. **B** qRT-PCR analysis the level of selected mRNAs in CFs upon silencing of IMP2 and Airn. **C** RIP and RT-PCR assays are conducted using anti-IMP2 to verify the binding of IMP2 with p53. **D** RNA FISH and immunofluorescence co-staining was employed to detect the colocalization of IMP2 protein (red), p53 mRNA (green) and DAPI (blue); upper scale bar = 20 μm, lower scale bar = 5 μm. **E** Representative images of immunofluorescence staining for EdU (green) and DAPI (bule) and quantification of the proliferative cells; Scale bar = 50 μm. **F** Representative images of immunofluorescence staining for α-SMA (green) and DAPI (blue) and quantification of the relative cell area of CFs; Scale bar = 50 μm. g Representative blot images and quantitative analysis of α-SMA, collagen I, p21, p53, Cyclin D1, CDK2 and CDK4 expression in CFs. **H** Representative blot images and quantitative analysis of p21, p53, Cyclin D1, CDK2 and CDK4 expression in CFs. **I** Flow cytometry analysis of cell cycle distribution. Data are presented as means ± SEM. *p < 0.05; **p < 0.01. n = 3 wells

As an m6A-binding protein, IMP2 is essential for mRNA stability. To check whether *Airn* could regulate IMP2-mRNA-binding, RIP-chip assay data with anti-IMP2 antibodies in *Airn* knockdown were obtained from the GSE87221 dataset in the GEO database. Six representative mRNAs associated with fibrosis (Gadd45b, Smad5, Mapk7, Mmmp14, Tcf7l1, and p53) were found to bind to IMP2 (Fig. 6A). We proposed that *Airn* may assist in the binding of IMP2 to mRNAs and may even affect the expression levels of these candidates. We then measured the mRNA expression levels of the six candidates after knocking down *Airn* and IMP2. The results showed that only p53 mRNA exhibited the same decreasing trend in response to *Airn* and IMP2 knockdown (Fig. 6B). Meanwhile, RIP and qRT-PCR assays identified that IMP2 did bind to p53 mRNA (Fig. 6C); thus, it was selected for further analysis. Immunofluorescence images revealed the colocalization of IMP2 protein with p53 mRNA in the cytoplasm (Fig. 6D). p53 is a negative regulator of fibrogenesis. However, the biological function of p53 in CFs from diabetic patients remains unclear. Western blotting results showed increased p53 expression in the heart tissues of diabetic mice, which is consistent with previous findings [41] (Additional file 5: Fig. S4A). Interestingly, the patterns of p53 expression in cardiomyocytes and CFs in response to HG were different, as p53 was upregulated in cardiomyocytes but downregulated in CFs after HG treatment (Additional file 5: Fig. S4B and C). As shown in Additional file 5: Fig. S4E, inhibiting p53 expression promoted the levels of α-SMA and collagen I in CFs cultured in NG, whereas p53 overexpression downregulated α-SMA and collagen I levels induced by HG treatment. Next, we examined whether p53 was a downstream target of *Airn*. Results of EDU staining and immunofluorescence for α-SMA showed that p53 knockdown significantly restored the proliferation and FMT of CFs with *Airn* overexpression and HG treatment (Fig. 6E and F). Meanwhile, Western blotting showed that p53 knockdown blunted the inhibitory effects of *Airn* on fibrosis, as evidenced by the enhanced fibrosis markers (α-SMA and collagen I) (Fig. 6G). As cell cycle progression and proliferation are regulated by p53, we propose that *Airn* may inhibit the activation of CFs by promoting p53-mediated cell cycle arrest. Moreover, HG treatment triggered downregulation of p21 and p53 and upregulation of cyclin-dependent kinases (cyclin D1, CDK2, and CDK4), which were abolished by *Airn* overexpression (Fig. 6G). However, the suppression of p53 inhibited the inhibitory effects of *Airn* on HG treatment. Next, we detected the effects of *Airn* on cell cycle distribution by Western blotting and flow cytometry. Western blotting results showed that *Airn* rescued the HG-mediated decrease in p53 levels. Consistent with the increased p53 expression, *Airn* triggered the upregulation of p21 and downregulation of cyclin D1, CDK2, and CDK4 (Fig. 6H). *Airn* silencing showed opposite trends in the expression of p53 and its downstream molecules. Flow cytometry demonstrated a decrease in the number of cells in the G1 and G2/M phases in the HG group, whereas the proportion of cells in the S phase increased markedly, suggesting increased proliferation of CFs. However, *Airn* overexpression significantly arrested the activated proliferation induced by HG. When *Airn* was silenced in the NG group, the number of cells in the S phase increased, whereas those in the G1 and G2/M phases declined significantly (Fig. 6I). These results show that *Airn* was sufficient to inhibit CF proliferation and FMT, thus alleviating cardiac fibrosis by regulating the p53-mediated cell cycle pathway.

It is well known that TGF-β signaling has a pivotal role in fibrosis, especially smads-dependent pathway (31531112, 35906830, 33995055). To further gain insight into the relationship between *Airn* and TGF-β/smads pathway, a serie of experiments were performed. We used disitertide, an inhibitor of TGF-β1, and exogenous TGF-β1 to inhibit or activate TGF-β1 for detecting the effects of TGF-β1 on *Airn* and IMP2 expression. The results of qPCR and western blotting showed that the altered level of TGF-β1 did not affect the levels of *Airn* and IMP2 (Additional file 4: Fig. S3A and B). Next, under NG condition, downregulated TGF-β1 did not reverse the positive effects of siAirn on collagen secretion and a-SMA expression (Additional file 4: Fig. S3C-L). In addition, *Airn* knockdown did not activated the crucial components of TGF-β pathways–smad2/3 (Additional file 4: Fig. S3C, I and J), which means that silencing *Airn* promotes fibrotic progress in TGF-β/smad2/3-independent manner. These results showed that *Airn* suppressed the fibrotic progress via regulating p53-mediated cell cycle rather than via affecting TGF-β/smads pathway.

### *Airn*/IMP2 stabilizes p53 mRNA in an m6A-dependent manner.

**Fig. 7:**
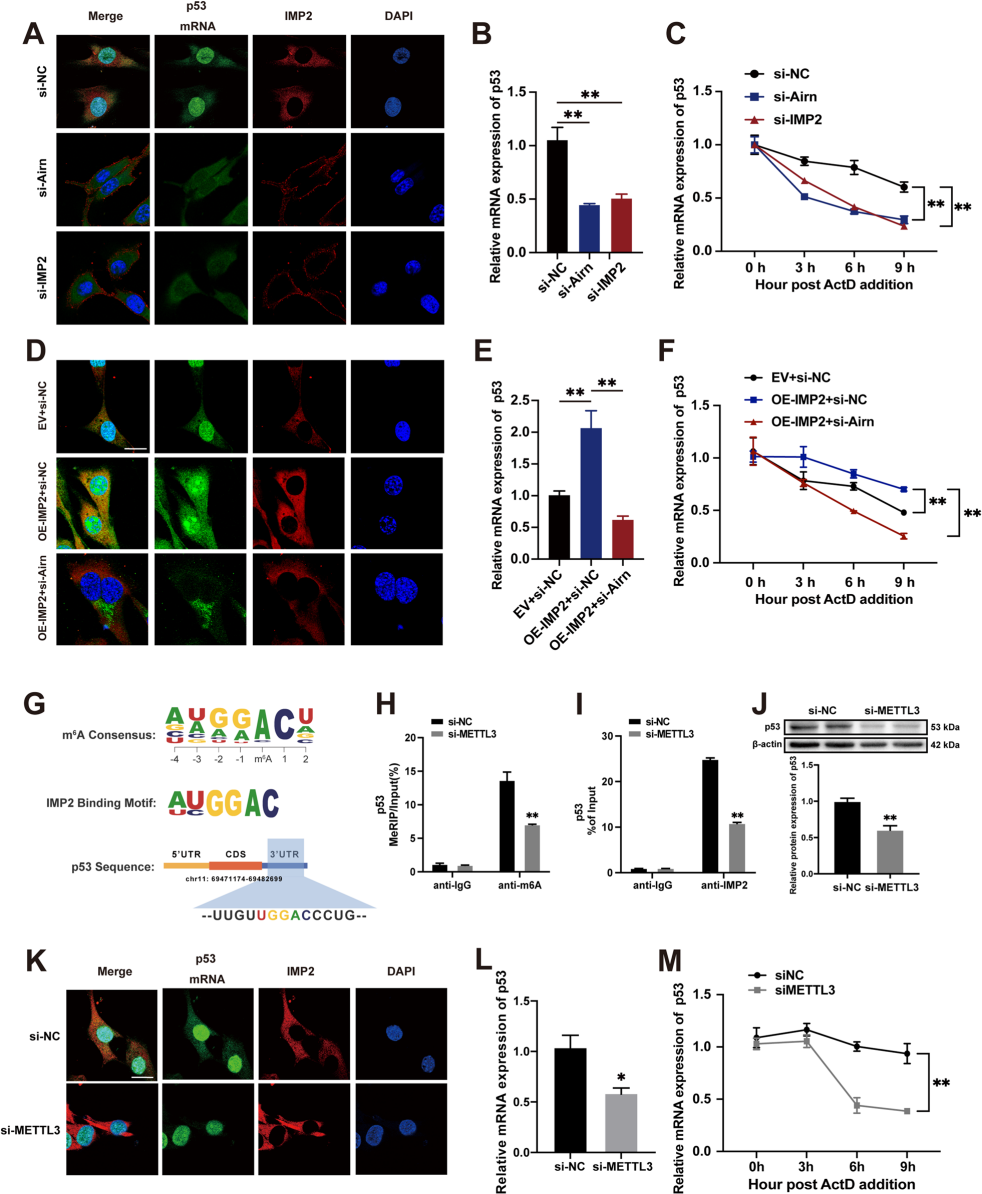
Airn/IMP2 stabilized p53 mRNA in an m6A-dependent manner. **A**, **D**, **K** RNA FISH and immunofluorescence co-staining was employed to detect the colocalization of IMP2 protein (red), p53 mRNA (green) and DAPI (blue); Scale bar = 20 μm. **B**, **E**, **L** qRT-PCR analysis of p53 mRNA in CFs. **C**, **F**, **M** The half-life of p53 mRNA in CFs were quantified by qRT-PCR at indicated time points after actinomycin D treatment in CFs. **G** Top consensus sequence of IMP2-binding sites and the m6A motif and predicted m6A-binding sequences are present in the p53 3’UTR region. **H** Enrichment of m6A modification in p53 wrote by METTL3 is detected by a gene-specific m6A qPCR assay. **I** RIP and RT-PCR assays were conducted using anti-IMP2 to verify the binding between IMP2 and p53 mRNA upon silencing METTL3. **J** Representative blot images and quantitative analysis of p53 upon silencing METTL3. Data are presented as means ± SEM. *p < 0.05; **p < 0.01. n = 3 wells

Previous studies have shown that IMP2 plays an important role in mRNA stabilization by binding to m6A-mRNAs [39]. We hypothesized that *Airn*/IMP2 regulates p53 by affecting p53 mRNA stabilization. Therefore, we performed double-label immunofluorescence and RNA decay assays to determine the stability of p53 mRNA. The double-label immunofluorescence assay showed that *Airn* knockdown inhibited the expression of IMP2 and p53 mRNA and weakened the binding of IMP2 and p53 mRNA, which is consistent with the si-IMP2 findings (Fig. 7A). RNA decay assays and qRT-PCR showed decreased expression and stability of p53 mRNA in CFs treated with si-*Airn* and si-IMP2, respectively (Fig. 7B and C). Consistent with these results in vitro, downregulated *Airn* also inhibited the stabilization of p53 mRNA (Additional file 6: Fig. S5G) and expression of p53 protein (Additional file 6: Fig. S5B and C) in fibroblasts from the heart of AAV9-sh*Airn* mice. Next, we examined whether *Airn* is a cofactor of IMP2 that regulates p53 expression. Exogenous overexpression of IMP2 enhanced binding with p53 mRNA and thus elevated the mRNA level of p53 (Fig. 7D and E). However, even when IMP2 was overexpressed, binding of IMP2 to p53 mRNA was clearly blunted by treatment with si*-Airn* (Fig. 7D). Therefore, *Airn* plays an essential role in the binding between IMP2 and p53 mRNA. Consistent with the results of double-label immunofluorescence, the weakened binding between IMP2 and p53 mRNA induced by si-*Airn* resulted in p53 mRNA instability in CFs with upregulation of IMP2 (Fig. 7F). Taken together, the above results suggest that *Airn* could contribute to the binding of IMP2 with p53 mRNA and synergistically work with IMP2 to maintain the stability of p53 mRNA.

To evaluate whether m6A modification is functionally important for IMP2-mediated p53 mRNA stabilization, we transfected CFs with si-METTL3 (methyltransferase-like protein 3), a core methyltransferase of the N6-adenosine-methyltransferase complex, which is known to modulate multiple biological processes [42, 43]. As reported previously, the consensus sequence ‘UGGAC’ motif is an m6A core motif enriched in the targets of the IMP family (IMP1, 2, and 3) [39] (Fig. 7G). The predicted potential binding site of p53 for IMP2 localization was in the 3’UTR, which coincides with the m6A distribution patterns (Fig. 7G). Upon METTL3 silencing, m6A modification of p53 decreased compared to that in the negative control group, as evidenced by the results of Me-RIP-PCR (Fig. 7H). The RIP assay demonstrated that IMP2 bound to enriched m6A-modified p53, and the binding between p53 and IMP2 was impaired upon METTL3-knockdown (Fig. 7I), suggesting that the recognition of p53 by IMP2 was based on m6A modifications. Meanwhile, decreased m6A modification by knocking down METTL3 triggered a decline in the expression of p53 at the protein level (Fig. 7J). More importantly, similar to the results of knocking down IMP2 and *Airn*, the effects of METTL3 downregulation on p53 showed a reduction in the stability of p53 mRNA and its subsequent levels in CFs (Fig. 7K–M). Overall, our data showed that m6A modifications are required for the binding of IMP2 to p53 mRNA and IMP2-mediated regulation of p53 expression.

## Discussion

Epidemiological findings suggest a strong relationship between diabetes and CVDs [44]. Diabetic cardiomyopathy, a well-known complication of diabetes, is characterized by microvascular damage, cardiomyocyte hypertrophy, and cardiac fibrosis. Intra-myocardial fibrosis is the main contributor to the reduction of myocardial compliance and impaired cardiac function. Although emerging evidence highlights the pivotal role of lncRNAs in the pathogenesis of cardiac fibrosis [45, 46], their underlying mechanisms remain unclear. In this study, we demonstrated that *Airn*, a CF-enriched lncRNA, could alleviate cardiac fibrosis by stabilizing p53 mRNA in an m6A-IMP2-dependent manner. First, we validated that *Airn* expression decreased significantly in heart tissues of diabetic mice and HG-treated CFs, which was accompanied by elevated cardiac fibrosis and reduced cardiac function. Second, overexpression of *Airn* suppressed diabetes-induced CF activation, leading to decreased fibrosis and consequently improved cardiac function, while knocking down *Airn* showed opposite trends. Mechanistically, Arin was bound to IMP2 and impeded its ubiquitination-dependent degradation. Preserved IMP2 promoted p53 mRNA stabilization and consequent protein expression through the identification of the m6A-methylated region, which ultimately prevented the activation of CFs and alleviated cardiac fibrosis in the heart tissues of diabetic mice. Thus, we propose a novel function of *Airn* in DCM fibrosis, thereby constituting a bridge between the epigenetic network of lncRNA *Airn* and m6A methylation in CVDs.

Studies on both clinical subjects and pre-clinical models of diabetes confirm the strong association between diabetes and cardiac fibrosis, which is accompanied by the devastation of myocardial architecture and functional deterioration [47]. Although previous studies elucidate multiple molecular mechanisms underlying cardiac fibrogenesis, a specific strategy for targeting cardiac fibrosis is lacking. Accumulating evidence suggests that lncRNAs are dynamically expressed in different CVDs and serve critical regulatory roles in gene expression. Notably, a recent study reports lncRNAs as potential mediators in pathological processes underlying cardiac fibrosis [48–53]. For instance, lncRNA-H19 is a competing endogenous RNA (ceRNA) for sponging miR-455; it increases miR-455-targeted gene CTGF to promote fibrosis [54]. Additionally, Wisper [55], CHRF [56], AK081284 [57], MEG3 [58] and MIAT [59] are also important pro-fibrotic lncRNAs in cardiac interstitial fibrosis and act via diverse regulatory mechanisms. Moreover, lncRNAs reportedly exert cardioprotective roles in the pathogenesis of cardiac fibrosis. Overexpression of GAS5 in TGF-β1-treated CFs inhibits proliferation and FMT of CFs by reducing miR-21 levels, thereby decreasing MMP-2 [60]. Although biological functions of the above-mentioned lncRNAs have been elucidated, the profound and multifaceted functions of a substantial proportion of lncRNAs remain unclear. Herein, the lncRNA *Airn* was enriched in the heart and was found to be downregulated in cardiac tissues of diabetic mice and high glucose-treated CFs. A previous study demonstrates the novel function of *Airn* in cardiomyocytes [35]. *Airn* knockdown reduces the survival of cardiomyocytes under cellular stress and enhances apoptosis. In hepatocellular carcinoma (HCC), *Airn* is highly expressed and its knockdown inhibits the progression of HCC by restraining cell proliferation and promoting apoptosis [32]. Decreased *Airn* expression is reported in diabetic nephropathy, thus leading to decreased cell viability and increased apoptosis. Moreover, forced expression of *Airn* exerts protective effects against diabetic nephropathy [31]. *Airn* downregulation is a contributor to cell apoptosis and an inhibitor in tumor cell proliferation; however, these findings are apparently contradictory to our results. We speculate that *Airn* plays multiple roles in different cells via different mechanisms. Specifically, the roles of *Airn* in inhibiting apoptosis of cardiomyocytes and activation of CFs are beneficial for cardiac remodeling. Therefore, *Airn* is a potential novel candidate for treatments targeting DCM fibrosis owing to its dual effects on cardiomyocytes and CFs.

Epigenetic modification regulates gene expression without changes in the DNA sequence, thus affecting several biological processes. N6-methyladenosine (m6A), the most common epigenetic modification of RNA, participates in cardiovascular homeostasis and diseases by influencing the stabilization, splicing, export, and translation of RNA [9–13]. Emerging evidence highlights the importance and therapeutic potential of m6A modifications in cardiac fibrosis [9, 10]. As an important m6A methylase, METTL3-mediated m6A modification regulates pathological processes underlying cardiac fibrosis. Overexpression of METTL3 activates the TGF-β/Smad2/3 pathway, thus mediating increased synthesis of ECM [17]. The m6A demethylase, FTO, decreases m6A methylation, thus effectively reducing cardiac fibrosis in myocardial infarction models [10]. These results indicated the vital role of m6A modifications in cardiac fibrosis. Given the role of methylases and demethylases in fibrogenesis, further investigations should focus on the regulatory effects of m6A readers, which directly initiate a series of biological processes in an m6A-dependent manner [10, 61, 62]. In this study, for the first time, we showed that IMP2, an important reader, decreased significantly in heart of diabetic mice. HG treatment promoted ubiquitination-dependent degradation of IMP2, leading to decreased stability of IMP2 and increased myocardial fibrosis. In contrast, IMP2 reconstitution in HG-treated CFs inhibited the progress of fibrosis by affecting the stabilization of its target genes. In line with our results, IMP2 participates in processes of various diseases by binding to several mRNAs, thus affecting the stabilization, transport, and translation of numerous genes [39]. Moreover, we provided clear evidence that lncRNA *Airn* was physically bound to IMP2 and impeded its degradation. Although the interaction of *Airn* and IMP2 has been reported in cardiomyocytes, our study, for the first time, elucidated the underlying mechanism by which *Airn* influences the stability of IMP2. In addition, we constructed IMP2 ubiquitin mutant plasmids (K77R and K139R) as reported previously [18] and found that *Airn* suppressed the ubiquitination of IMP2 by masking the K139 ubiquitin site, thus slowing its degradation under high-glucose stimulation. Taken together, our results indicate that the lncRNA/m6A reader/mRNA axis in CFs is responsible for the development of diabetes-induced cardiac fibrosis.

The role of IMP2 has been extensively studied in various cancers [18, 63, 64]. IMP2 facilitates the growth and metastasis in several cancers by enhancing the mRNA stability or translation of oncogenic factors [18–20]. However, the function and mechanisms of IMP2 remain unclear in CVDs, especially in DCM. Consistent with the functions of *Airn*, downregulation of IMP2 promoted collagen secretion in CFs treated with HG. Our findings show that HG facilitates the ubiquitination of IMP2 in CFs and *Airn* plays a protective role in preventing the degradation of IMP2 by ubiquitination. Therefore, we elucidate a previously unrecognized mechanism of IMP2 degradation and a novel function of *Airn* in IMP2 ubiquitination.

p53 reduced the activation of CFs triggered by HG. Previous studies have confirmed that p53 plays a critical regulatory role in fibrosis. However, the role and mechanism of p53 in the progression of cardiac fibrosis remain unclear. In the cardiac senescence model, p53 prevented the development of cardiac fibrosis by inhibiting the cyclin protein, thus blocking the cell cycle and suppressing the proliferation of myofibroblasts [65]. Moreover, forced expression of p53 impairs the proliferative capacity of CFs and protects against fibrosis in failing hearts [66]. Consist with these results, our findings demonstrated diabetes-induced p53 inhibition in CFs. Restoring p53 by promoting its mRNA stability prevents the proliferation of CFs and further protects against diabetes-induced cardiac fibrosis. Interestingly, p53 levels were high in the heart tissues of diabetic mice and cardiomyocytes exposed to HG, which has been reported in clinical heart tissues of diabetic patients as well. Considering the vigorous proliferation potential of CFs and the well-established role of p53 in cell proliferation, inhibition of p53 expression in CFs may play a more vital role in the pathogenesis of diabetes-induced cardiac fibrosis, and further studies are needed to examine the role of p53 elevation in diabetes-induced cardiomyocytes injury. Another important finding was that IMP2 was bound to m6A-modified p53 mRNA, thus further maintaining its mRNA stabilization and protein expression. Based on the important role of p53 in the cell cycle, we provide novel insight into the roles of the lncRNA-p53 axis in cell cycle arrest, through which *Airn* inhibits CF activation. Additionally, m6A modification is also essential for p53 mRNA stabilization and expression via METTL3 methylation and IMP2 recognition. However, a previous study suggests that hypomethylation increases p53 expression after METTL3 knockdown in the leukemic cell line, K562 [67]. Interestingly, another investigation proposes an opposite conclusion that mRNA and protein levels of p53 remain significantly unchanged by siMETTL3 in the human keratinocyte lines [68]. It is therefore controversial how m6A modification regulates the expression of p53. m6A influences the fate of mRNA by recruiting specific m6A readers. Due to the vast heterogeneity of cell and tissue types, the effects of methylation on translation and stability of certain mRNAs depend on which reader is dominant under the specific cellular context [69]. Thus, we identified a novel regulatory mechanism of p53 in DCM fibrosis, whereby *Airn* synergistically functions with m6A modifications to stabilize p53 and enhances its expression.

However, this study has some limitations. First, we did not analyze heart tissue from biopsies of diabetic patients for ethical reasons. Second, we only investigated the mechanisms of *Airn* in animal models of type I diabetes. However, Type II diabetes was more common than type I clinically. Further study is required to investigate the role of *Airn* in heart of type II diabetes. Third, in this study, we only focused on the effects of *Airn* on CFs since CFs are the main source of cardiac fibrosis. According to the beneficial effects of *Airn* overexpression on cardiac functions, we believe that *Airn* may regulate biological processes of cardiomyocytes and affect cardiac function, and this needs to be studied further. Fourth, *Airn* could regulate the expression of p53 in an m6A-IMP2-dependent manner. Given widespread m6A modifications in diabetic heart tissues [16], further studies are needed to examine the effects of *Airn* on other m6A-modified mRNAs. Fifth, we did not investigate the regulatory role of HG on *Airn*, which is important for treatment of DCM-related fibrosis via targeting *Airn*. Despite these limitations, our data provide strong evidence on the roles of *Airn*-manipulated proliferation and FMT of CFs along with fibrosis in DCM.

## Conclusion

In summary, we revealed a novel mechanism by which lncRNA *Airn* prevented IMP2 degradation and played a synergetic role with IMP2 in promoting p53 mRNA stabilization in an m6A modification-dependent manner, thus, alleviating myocardial fibrosis in DCM. Our results provide new insights for understanding the essential role of *Airn* as a co-factor in epigenetic modulation in an anti-fibrotic manner. *Airn* is potentially useful for the development of lncRNA-based therapies for cardiac fibrosis.

## Supplementary Information

**Additional file 1. **The IMP2 protein sequences.

**Additional file 2. Fig. S1.** Airn is downregulated in diabetic mice heart tissues.

**Additional file 3. Fig. S2. **Depletion of Airn resulted in the decline of IMP2 at the protein level but not mRNA level.

**Additional file 4. Fig. S3. **Airn effected the fibrotic progress independent of TGF-β/smads signaling pathway in HG-treated CFs.

**Additional file 5. Fig. S4. **p53 exerts an important negative role in HG-induced fibrosis in vitro.

**Additional file 6. Fig. S5. **Cardiac fibroblasts isolated form the heart in CTRL+AAV9-shAirn mice share the same the cellular mechanisms in vitro.

## Data Availability

The results of the manuscript are based on the relevant datasets available in the manuscript.

## Abbreviations

DCM: Diabetic cardiomyopathy
STZ: Streptozotocin
AAV9: Serotype 9 adeno-associated virus
CFs: Cardiac fibroblasts
HG: High glucose
NG: Normal glucose
IMP2: Insulin-like growth factor 2 mRNA-binding protein 2
FMT: Fibroblast-to-myofibroblast transition
m6A: N6-methyladenosine
CVDs: Cardiovascular diseases
WT: Wild-type
WB: Western blotting
qRT-PCR: Quantitative real-time PCR
FISH: RNA fluorescence in situ hybridization
IF: Immunofluorescence
RIP: RNA immunoprecipitation
Me-RIP: M6A-RNA immunoprecipitation
IP: Immunoprecipitation
IHC: Immunohistochemical analyses
EdU: 5-Ethynyl-2’-deoxyuridine
CHX: Cycloheximide
Act-D: Actinomycin D
CTRL: Control
LVEF: Left ventricular ejection fraction
LVFS: Left ventricular fractional shorting
LVIDs: Left ventricular systolic internal dimension
LVIDd: Left ventricular diastolic internal dimension
