# Supplementary material for: LncRNA Airn alleviates diabetic cardiac fibrosis by inhibiting activation of cardiac fibroblasts via a m6A-IMP2-p53 axis
Source: Biol Direct. 2022 Nov 16;17:32. doi: 10.1186/s13062-022-00346-6 (PMC9670606; doi:10.1186/s13062-022-00346-6)
Supplement: Supplementary file 1 — Additional file 1. The IMP2 protein sequences. [file 13062_2022_346_MOESM1_ESM.docx]

**Additional file**

# The IMP2 protein sequences

# [Homo sapiens](https://www.ncbi.nlm.nih.gov/Taxonomy/Browser/wwwtax.cgi?mode=Info&id=9606)

1 mmnklyignl spavtaddlr qlfgdrklpl agqvllksgy afvdypdqnw airaietlsg

61 kvelhgkime vdysvskklr srkiqirnip phlqwevldg llaqygtven veqvntdtet

121 avvnvtyatr eeakiamekl sghqfenysf kisyipdeev sspsppqraq rgdhssreqg

181 hapggtsqar qidfplrilv ptqfvgaiig kegltiknit kqtqsrvdih rkensgaaek

241 pvtihatpeg tseacrmile imqkeadetk laeeiplkil ahnglvgrli gkegrnlkki

301 ehetgtkiti sslqdlsiyn pertitvkgt veacasaeie imkklreafe ndmlavnths

361 gyfsslyphh qfgpfphhhs ypeqeivnlf iptqavgaii gkkgahikql arfagasiki

421 apaegpdvse rmviitgppe aqfkaqgrif gklkeenffn pkeevkleah irvpsstagr

481 vigkggktvn elqnltsaev ivprdqtpde neevivriig hffasqtaqr kireivqqvk

541 qqeqkypqgv asqrsk

# Mus musculus

1 mmnklyignl spavtaddlr qlfgdrklpl agqvllksgy afvdypdqnw airaietlsg

61 kvelhgkime vdysvskklr srriqirnip phlqwevldg llaeygtven veqvntdtet

121 avvnvtymtr eeaklaiekl sghqfedysf kisyipdeev sspspphrar eqghgpgsss

181 qarqidfplr ilvptqfvga iigkegltik nitkqtqsrv dihrkensga aekpvtihat

241 pegtseacrm ileimqkead etklaeevpl kilahngfvg rligkegrnl kkiehetgtk

301 itisslqdls iynpertitv rgtieacana eieimkklre afendmlavn qqanlipgln

361 lsalgifstg lsvlpppagp rgvppsppyh pfathsgyfs slyphhhfgp fphhhsypeq

421 etvslfiptq avgaiigkkg ahikqlarfa gasikiapae gpdvsermvi itgppeaqfk

481 aqgrifgklk eenffnpkee vkleahirvp sstagrvigk ggktvnelqn ltsaevivpr

541 dqtpdeneev ivriighffa sqtaqrkire ivqqvkqqeq rypqgvapqr sk

**Rattus norvegicus**

1 mmnklyignl spavtaddlr qlfgdrklpl agqvllksgy afvdypdqnw airaietlsg

61 kvelhgkime vdysvskklr srkiqirnip phlqwevldg llaeygtven veqvntdtet

121 avvnvtymtr eeakvaiekl sghqfedysf kisyipdeel sspspphrar eqghgpgsss

181 qarqidfplr ilvptqfvga iigkegltik nitkqtqsrv dihrkensga aekpvtihat

241 pegtseacrm ileimqkead etklaeevpl kilahngfvg rligkegrnl kkiehetgtk

301 itisslqdls iynpertitv kgtieacasa emeimkklre afendmlavn qqanlipgln

361 lsalgifstg lsvlpppagp rgappsppyh pfathsgyfs slyphhhfgp fphhhsypeq

421 etvslfiptq avgaiigkkg ahikqlarfa gasikiapae gpdvsermvi itgppeaqfk

481 aqgrifgklk eenffnpkee vkleahirvp sstagrvigk ggktvnelqn ltsaevivpr

541 dqtpdeneev ivriighffa sqtaqrkire ivqqvkqqaq kypqgvapqr sk
