# Supplementary material for: LncRNA Airn alleviates diabetic cardiac fibrosis by inhibiting activation of cardiac fibroblasts via a m6A-IMP2-p53 axis
Source: Biol Direct. 2022 Nov 16;17:32. doi: 10.1186/s13062-022-00346-6 (PMC9670606; doi:10.1186/s13062-022-00346-6)
Supplement: Supplementary file 2 — Additional file 2. Fig. S1. Airn is downregulated in diabetic mice heart tissues. [file 13062_2022_346_MOESM2_ESM.docx]

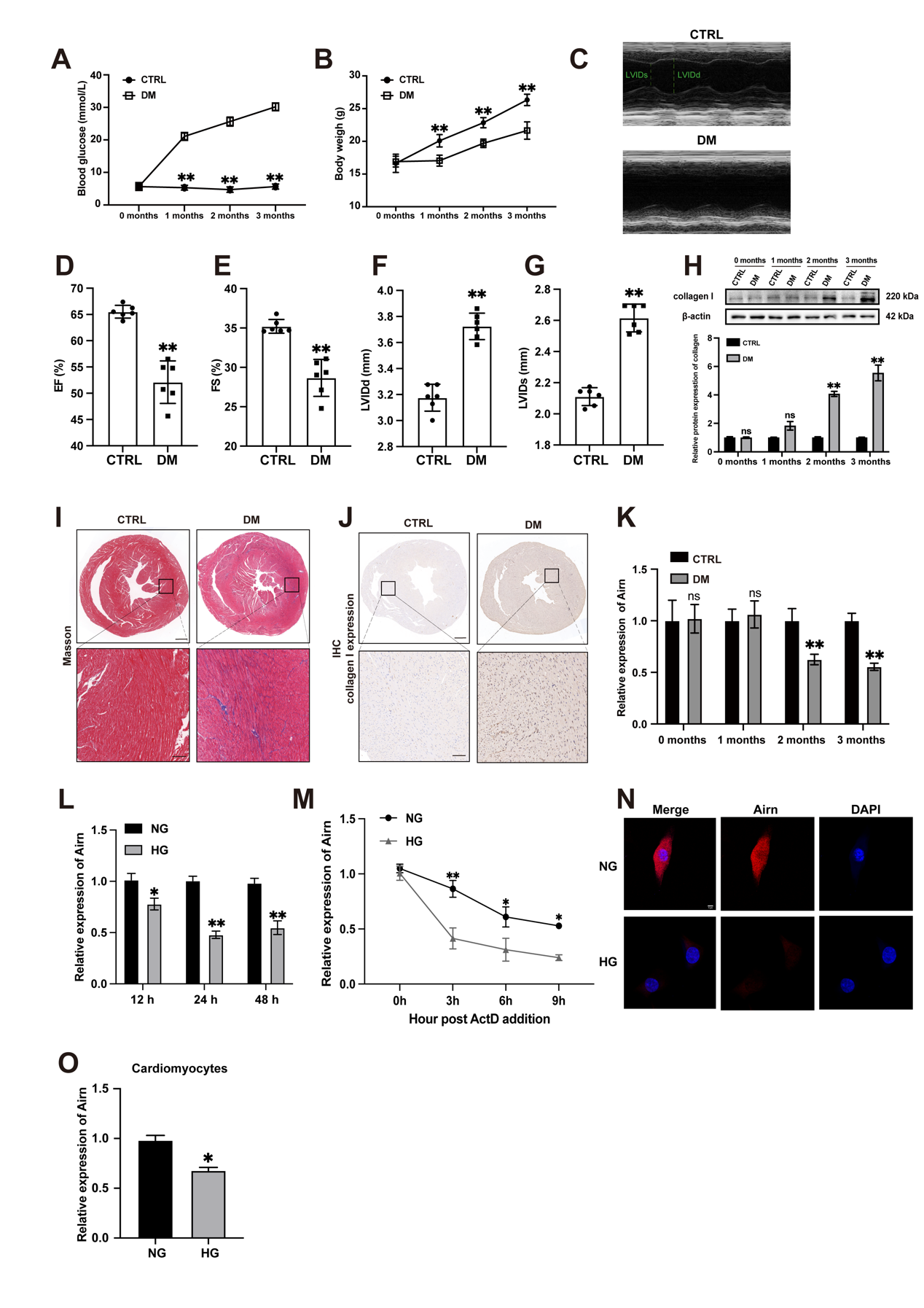


Fig. S1 Airn is downregulated in diabetic mice heart tissues. (a) Blood glucose of control and diabetic mice were determined every 1 month. (b) Body weight of control and diabetic mice were determined every 1 month. (c) Representative M-mode echocardiography images obtained from CTRL mice and DM mice at 3 months after STZ injection; LVIDd and LVIDs are labelled. (d-e) Echocardiography data analysis. (h) Representative blot images and quantitative analysis of collagen I expression. (i) Representative images of Masson’s trichrome staining in the hearts of CTRL mice and DM mice at 3 months after STZ injection; upper scale bar = 2 mm, lower scale bar = 50 μm. (j) Representative immunohistochemical stains of Collagen I in the hearts of CTRL mice and DM mice at 3 months after STZ injection; upper scale bar = 2 mm, lower scale bar = 50 μm. (k) qRT-PCR analysis of Airn in CTRL and DM hearts at indicated time points. (l) qRT-PCR analysis of Airn in CFs. (m) The half-life of Airn were quantified by qRT-PCR at indicated time points after actinomycin D treatment in CFs. (n) RNA FISH analysis was employed to detect the expression in CFs; Scale bar = 20 μm. (o) qRT-PCR analysis of Airn in cardiomyocytes. Data are presented as means ± SEM. **p < 0.01. n=6 mice or 3 wells.
