# Supplementary material for: LncRNA Airn alleviates diabetic cardiac fibrosis by inhibiting activation of cardiac fibroblasts via a m6A-IMP2-p53 axis
Source: Biol Direct. 2022 Nov 16;17:32. doi: 10.1186/s13062-022-00346-6 (PMC9670606; doi:10.1186/s13062-022-00346-6)
Supplement: Supplementary file 3 — Additional file 3. Fig. S2. Depletion of Airn resulted in the decline of IMP2 at the protein level but not mRNA level. [file 13062_2022_346_MOESM3_ESM.docx]

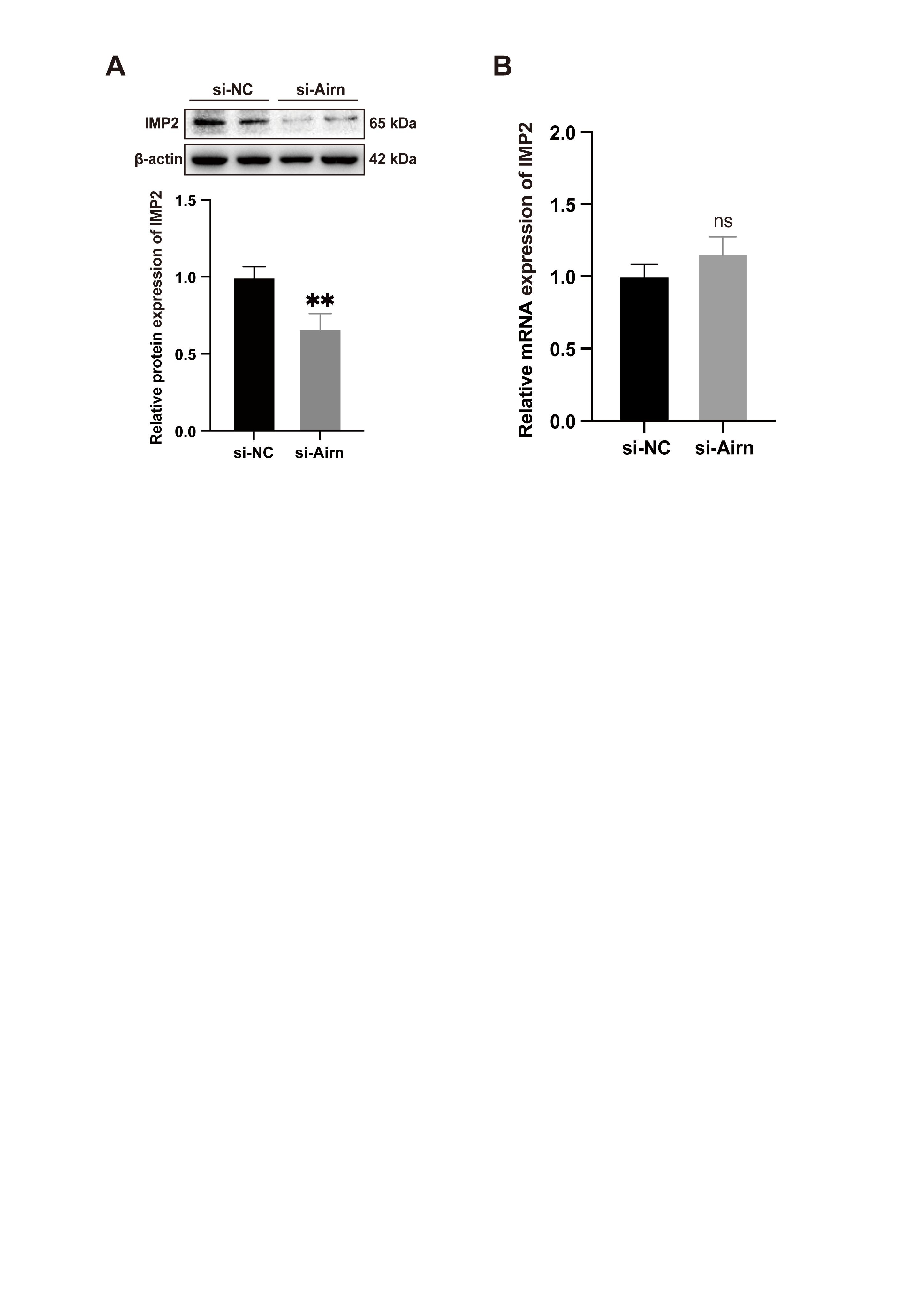


Fig. S2 Depletion of Airn resulted in the decline of IMP2 at the protein level but not mRNA level. Data are presented as means ± SEM. **p < 0.01. n= 3 wells.
