# Supplementary material for: LncRNA Airn alleviates diabetic cardiac fibrosis by inhibiting activation of cardiac fibroblasts via a m6A-IMP2-p53 axis
Source: Biol Direct. 2022 Nov 16;17:32. doi: 10.1186/s13062-022-00346-6 (PMC9670606; doi:10.1186/s13062-022-00346-6)
Supplement: Supplementary file 4 — Additional file 4. Fig. S3. Airn effected the fibrotic progress independent of TGF-β/smads signaling pathway in HG-treated CFs. [file 13062_2022_346_MOESM4_ESM.docx]

Fig. S3 Airn effected the fibrotic progress independent of TGF-β/smads signaling pathway in HG-treated CFs. (a) qRT-PCR analysis of Airn expression in CFs. (b) Representative blot images and the quantitative analysis of IMP2 in CFs. (c) Representative blot images. (d-j) The quantitative analysis of proteins expression. (k and l) Representative images and the quantitative analysis of immunofluorescence staining for α-SMA (red) and DAPI (blue); Scale bar = 50 μm. Data are presented as means ± SEM. *p < 0.05, **p < 0.01. n=3 wells.
