# Supplementary material for: LncRNA Airn alleviates diabetic cardiac fibrosis by inhibiting activation of cardiac fibroblasts via a m6A-IMP2-p53 axis
Source: Biol Direct. 2022 Nov 16;17:32. doi: 10.1186/s13062-022-00346-6 (PMC9670606; doi:10.1186/s13062-022-00346-6)
Supplement: Supplementary file 5 — Additional file 5. Fig. S4. p53 exerts an important negative role in HG-induced fibrosis in vitro. [file 13062_2022_346_MOESM5_ESM.docx]

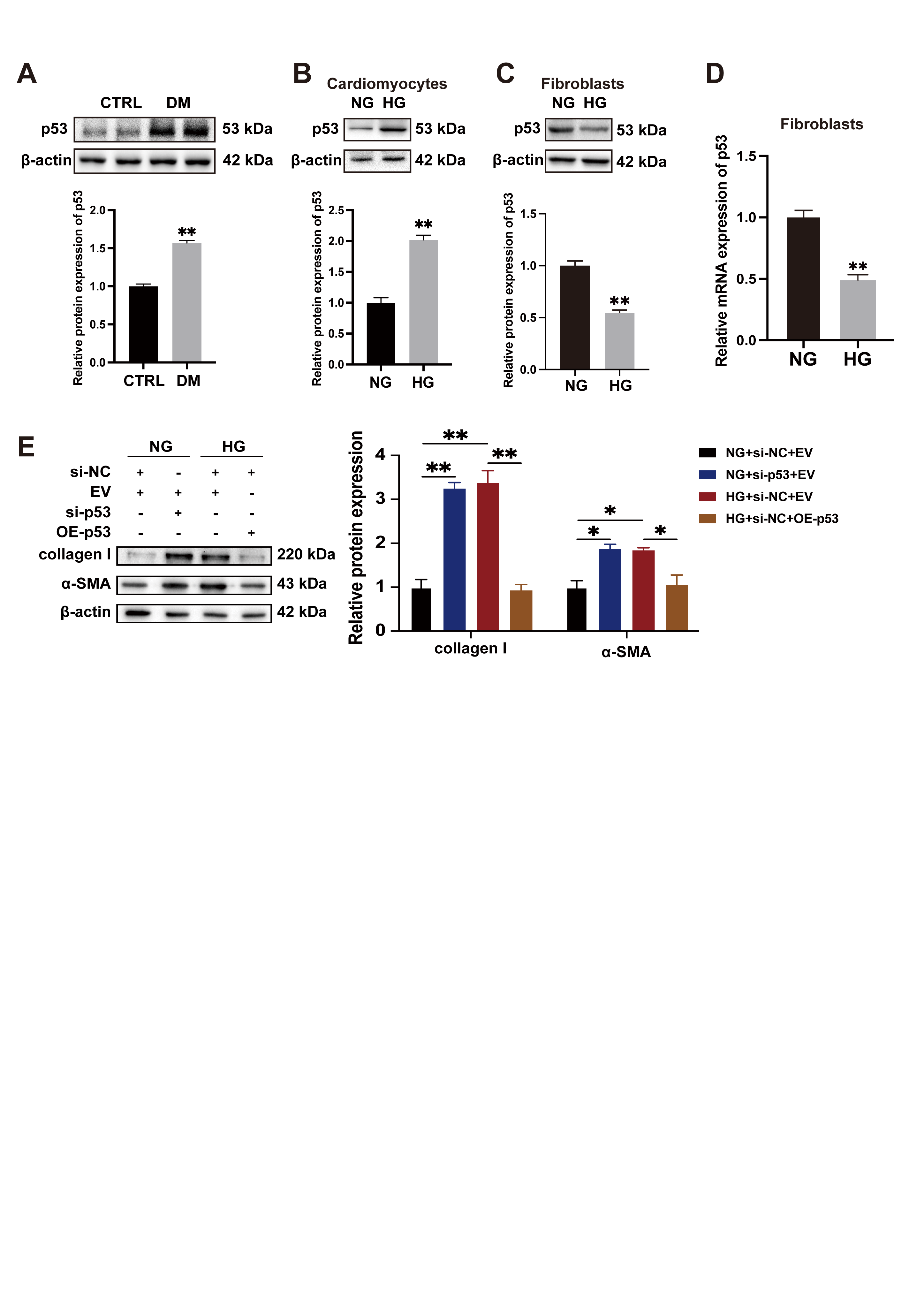


Fig. S4 p53 exerts an important negative role in HG-induced fibrosis in vitro. (a-c) Representative blot images and quantitative analysis of p53 expression in mice heart tissues, cardiomyocytes, and cardiac fibroblasts. (d) qRT-PCR analysis of p53 mRNA. (e) Representative blot images and quantitative analysis of p53 expression in CFs. Data are presented as means ± SEM. **p < 0.01. n=6 mice or 3 wells.
