# Supplementary material for: LncRNA Airn alleviates diabetic cardiac fibrosis by inhibiting activation of cardiac fibroblasts via a m6A-IMP2-p53 axis
Source: Biol Direct. 2022 Nov 16;17:32. doi: 10.1186/s13062-022-00346-6 (PMC9670606; doi:10.1186/s13062-022-00346-6)
Supplement: Supplementary file 6 — Additional file 6. Fig. S5. Cardiac fibroblasts isolated form the heart in CTRL+AAV9-shAirn mice share the same the cellular mechanisms in vitro. [file 13062_2022_346_MOESM6_ESM.docx]

Fig. S5 Cardiac fibroblasts isolated form the heart in CTRL+AAV9-shAirn mice share the same the cellular mechanisms in vitro. (a) IP and ubiquitination assays were conducted to investigate the effect of Airn on ubiquitination of IMP2 in vivo. (b-f) Representative blot images and quantitative analysis of proteins expression in CFs isolated form heart. (g) The half-life of p53 mRNA were quantified by qRT-PCR at indicated time points after actinomycin D treatment in CFs. (h) CCK-8 assay showed the proliferation of CFs. (i) Representative images of immunofluorescence staining for α-SMA (red) and DAPI (blue) of CFs; Scale bar = 50 μm. Data are presented as means ± SEM. *p < 0.05, **p < 0.01. n= 3 wells.
